# Supplementary material for: Association of maternal depression and anxiety with toddler social-emotional and cognitive development in South Africa: a prospective cohort study
Source: BMJ Open. 2022 Apr 13;12(4):e058135. doi: 10.1136/bmjopen-2021-058135 (PMC9014070; doi:10.1136/bmjopen-2021-058135)
Supplement: Author's manuscript [file bmjopen-2021-058135.draft_revisions.pdf]

BMJ Open is committed to open peer review. As part of this commitment we make the peer review history of every article we publish publicly available.

When an article is published we post the peer reviewers' comments and the authors' responses online. We also post the versions of the paper that were used during peer review. These are the versions that the peer review comments apply to.

The versions of the paper that follow are the versions that were submitted during the peer review process. They are not the versions of record or the final published versions. They should not be cited or distributed as the published version of this manuscript.

BMJ Open is an open access journal and the full, final, typeset and author-corrected version of record of the manuscript is available on our site with no access controls, subscription charges or pay-per-view fees (http://bmjopen.bmj.com).

If you have any questions on BMJ Open's open peer review process please email info.bmjopen@bmj.com

BMJ Open

Association of Maternal Depression and Anxiety with Toddler Social-Emotional and Cognitive Development in South Africa: A prospective cohort study

Journal:	*BMJ Open*
Manuscript ID	bmjopen-2021-058135
Article Type:	Original research
Date Submitted by the Author:	07-Oct-2021
Complete List of Authors:	Shuffrey, Lauren; Columbia University Irving Medical Center, Psychiatry; New York State Psychiatric Institute, Division of Developmental Neuroscience<br>Sania, Ayesha; Columbia University Irving Medical Center, Psychiatry; New York State Psychiatric Institute, Division of Developmental Neuroscience<br>Brito, Natalie; New York University, Department of Applied Psychology<br>Potter, Mandy; Stellenbosch University Faculty of Medicine and Health Sciences, Obstetrics and Gynaecology<br>Springer, Priscilla; Stellenbosch University, Paediatrics and Child Health<br>Lucchini, Maristella ; Columbia University Irving Medical Center, Psychiatry; New York State Psychiatric Institute, Neuroscience<br>Rayport, Yael; Columbia University Irving Medical Center, Department of Psychiatry; New York State Psychiatric Institute, Neuroscience<br>Du Plessis, Carlie; Stellenbosch University Faculty of Medicine and Health Sciences, Department of Obstetrics and Gynaecology<br>Odendaal, H. J.; Stellenbosch University Faculty of Medicine and Health Sciences, Obstetrics & Gynaecology<br>Fifer, William; Columbia University Irving Medical Center, Psychiatry; New York State Psychiatric Institute, Neuroscience
Keywords:	Community child health < PAEDIATRICS, Depression & mood disorders < PSYCHIATRY, Anxiety disorders < PSYCHIATRY, Child & adolescent psychiatry < PSYCHIATRY

SCHOLARONE™
Manuscripts

I, the Submitting Author has the right to grant and does grant on behalf of all authors of the Work (as defined in the below author licence), an exclusive licence and/or a non-exclusive licence for contributions from authors who are: i) UK Crown employees; ii) where BMJ has agreed a CC-BY licence shall apply, and/or iii) in accordance with the terms applicable for US Federal Government officers or employees acting as part of their official duties; on a worldwide, perpetual, irrevocable, royalty-free basis to BMJ Publishing Group Ltd ("BMJ") its licensees and where the relevant Journal is co-owned by BMJ to the co-owners of the Journal, to publish the Work in this journal and any other BMJ products and to exploit all rights, as set out in our licence.

The Submitting Author accepts and understands that any supply made under these terms is made by BMJ to the Submitting Author unless you are acting as an employee on behalf of your employer or a postgraduate student of an affiliated institution which is paying any applicable article publishing charge ("APC") for Open Access articles. Where the Submitting Author wishes to make the Work available on an Open Access basis (and intends to pay the relevant APC), the terms of reuse of such Open Access shall be governed by a Creative Commons licence – details of these licences and which Creative Commons licence will apply to this Work are set out in our licence referred to above.

Other than as permitted in any relevant BMJ Author's Self Archiving Policies, I confirm this Work has not been accepted for publication elsewhere, is not being considered for publication elsewhere and does not duplicate material already published. I confirm all authors consent to publication of this Work and authorise the granting of this licence.

For peer review only

Title: Association of Maternal Depression and Anxiety with Toddler Social-Emotional and Cognitive Development in South Africa: A prospective cohort study

Lauren C. Shuffrey*[1,2], Ayesha Sania*[1,2], Natalie H. Brito[3], Mandy Potter[4], Priscilla E. Springer[5], Maristella Lucchini[1,2], Yael Rayport[1,2], Carlie Du Plessis[4], Hein J. Odendaal[4], William P. Fifer[1,2,6]

[1] Department of Psychiatry, Columbia University Irving Medical Center, New York, NY 10032 USA
[2] Division of Developmental Neuroscience, New York State Psychiatric Institute, New York, NY 10032 USA
[3] Department of Applied Psychology, New York University, New York, NY, 10003 USA
[4] Department of Obstetrics and Gynaecology, Faculty of Medicine and Health Science, Stellenbosch University, Cape Town, Western Cape, South Africa 7530
[5] Department of Paediatrics and Child Health, Stellenbosch University, Cape Town, Western Cape, South Africa 7530
[6] Department of Pediatrics, Columbia University Irving Medical Center, New York, NY 10032 USA
*** denotes shared authorship**

Corresponding Author:
Lauren C. Shuffrey, PhD
Columbia University Medical Center
New York State Psychiatric Institute
1051 Riverside Drive, Unit 40
New York, New York 10032
lcg2129@cumc.columbia.edu

Financial Support: This research was supported by the Bill and Melinda Gates Foundation (WPF) and grants U01HD055154, U01HD045935, U01HD055155, and U01HD045991 issued by the *Eunice Kennedy Shriver* National Institute of Child Health and Human Development (NICHD) and grant U01AA016501 issued by the National Institute on Alcohol Abuse and Alcoholism (NIAAA). Dr. Lauren Shuffrey is supported by K99HD103910 issued by the Eunice Kennedy Shriver National Institute Of Child Health & Human Development. Dr. Ayesha Sania is supported by UH3OD023279-05S1, re-entry supplement from Office of the Director, NIH, and Office of Research on Women Health (ORWH). The content is solely the responsibility of the authors and does not necessarily represent the official views of the National Institutes of Health.

Competing Interests: The authors have no competing interests to declare.

Data availability: Data are available on request.

Patient and Public Involvement: Patients or the public were not involved in the design, or conduct, or reporting, or dissemination plans of our research

For peer review only

Abstract:

Objective: A robust literature has identified associations between prenatal maternal depression and adverse child social-emotional and cognitive outcomes. The majority of prior research is from high-income countries despite increased reporting of perinatal depression in low and middle income countries (LMICs). Additionally, despite the comorbidity between depression and anxiety, few prior studies have examined their joint impact on child neurodevelopment. The objective of the current analysis was to examine associations between prenatal maternal depression and anxiety with child social-emotional and cognitive development in a cohort from the Western Cape Province of South Africa.

Design: Prenatal maternal depression and anxiety were measured using the Edinburgh Postnatal Depression Scale (EPDS) and the State-Trait Anxiety Scale (STAI) at 20 – 24 weeks' gestation. Child neurobehavior was assessed at age 3 using the Brief Infant Toddler Social Emotional Assessment (BITSEA) and the Bayley Scales of Infant Development (BSID-III). We used linear regression models to examine the independent and joint association between prenatal maternal depression, anxiety, and child developmental outcomes.

Results: Participants consisted of 600 maternal-infant dyads (274 females; gestational age at birth: 38.89 weeks ± 2.03). Children born to mothers with both prenatal depression and trait anxiety had the largest increase in social-emotional problems (mean difference: 4.66; 95% CI 3.43, 5.90) compared to children born to mothers with no prenatal depression or trait anxiety, each condition alone, or compared to mothers with depression and state anxiety. Additionally, children born to mothers with prenatal maternal depression and trait anxiety had the greatest reduction in mean cognitive scores on the BSID-III (mean difference: -1.04; 95% CI -1.99, -0.08).

Conclusions: The observed association between comorbid prenatal maternal depression and chronic anxiety with subsequent child social-emotional and cognitive development underscores the need for targeting mental health support in perinatal women in LMICs to improve long-term child neurobehavioral outcomes.

Strengths and limitations of this study

- Strengths include prospective evaluation of maternal depression and anxiety symptoms during pregnancy and prospective assessment of cognitive and social-emotional outcomes within the same cohort in a large sample of mother-children pairs.

- Limitations include a lack of data on maternal mental health assessments postnatally and mother-child dyadic measures, which are potential mediators of the relationship between prenatal maternal depression, prenatal maternal anxiety, and child developmental outcomes.

For peer review only - http://bmjopen.bmj.com/site/about/guidelines.xhtml

Key Questions:

What is already known about this subject?

- Prenatal maternal depression and anxiety are associated with adverse child social-emotional and cognitive outcomes.
- The majority of prior research in this domain is from high-income countries.
- In South Africa an estimated 35% women report prenatal depression.

What does this study add?

- Our results from a South African cohort study suggest comorbid prenatal maternal depression and chronic anxiety have a greater impact on child social-emotional and cognitive development than either condition alone or than comorbid prenatal maternal depression and transitory anxiety during pregnancy.

How might this impact on clinical practice?

- These results underscore the need for targeting mental health in perinatal women in low and middle income countries to improve long-term child neurobehavioral outcomes.

For peer review only

Keywords: prenatal depression, prenatal anxiety; social-emotional development; cognitive development

Introduction

Decades of research on the early origins of behavior has promoted the concept that the prenatal environment has a profound impact on one's risk for the development of neurodevelopmental or psychiatric disorders[1 2]. Several prior studies have identified associations between prenatal maternal depression and increased risk for social-emotional problems and decrements in cognitive development. However, the majority of prior research in this domain is from high-income countries (HICs), despite increased reporting of perinatal depression in low and middle income countries (LMICs) including South Africa where an estimated 35% women report prenatal depression[3 4]. Additionally, despite the comorbidity between depression and anxiety, few prior studies have examined their joint impact on child neurodevelopment.

Several studies in HICs have identified associations between prenatal maternal depression, anxiety, and offspring behavioral, social-emotional, and cognitive development[5]. Specifically, a meta-analysis demonstrated adverse effects of prenatal maternal depression and anxiety on child social-emotional problems, with odds ratios of 1.79 and 1.50 respectively[6]. Prenatal maternal depression and anxiety are also associated with cognitive and language deficits[7 8], delayed motor development[8], emotional and behavior dysregulation[9-11], inattention and hyperactivity[12-14], and difficult temperament[15]. A more recent meta-analysis not only confirmed prior reports, but also found that the effects of perinatal maternal depression extend beyond infancy through adolescence[16].

The developmental origins of health and disease (DOHaD) model posits that maternal psychological distress during pregnancy (e.g. perceived stress, depression, anxiety, trauma) may result in changes in hypothalamic pituitary adrenal (HPA) axis function and upregulation of inflammatory processes with downstream effects on offspring perinatal brain development and

For peer review only - http://bmjopen.bmj.com/site/about/guidelines.xhtml

behavior[17]. These risk factors may be exacerbated in LMICs such as South Africa, where both poverty and perinatal mental health disorders are highly prevalent[18 19]. Specifically, in South Africa maternal mood disorders have been linked to structural and community stressors associated with markers of poverty including less than a high school education, lack of social support, alcohol use, family stress, food insecurity, lack of partner involvement, and intimate partner violence[20-24]. There are few prior studies that have examined the impact of prenatal maternal mental health on offspring social-emotional or cognitive development in South Africa. While the few prior studies reported significant harmful effects of prenatal maternal stress or perinatal maternal depression, there is still a significant gap in the literature in research examining the impact of prenatal maternal psychological health on child neurobehavioral development in resource-poor communities[20 25 26]. Additionally, to our knowledge no prior South African studies have examined the joint effect of prenatal maternal depression and anxiety on child neurobehavioral outcomes. Research examining the long-term impact of prenatal maternal depression and anxiety on child behavioral and cognitive outcomes is critical for providing justification to local public health services for targeting mental health support in perinatal women from underserved communities.

The objective of the current analysis was to determine if prenatal maternal depression and state or trait anxiety were associated with child social-emotional problems or cognitive development at approximately three years of age in a South African cohort from the Western Cape.

For peer review only

Materials and Methods

Participants. Participants were a subset of infants with available outcome data at age 3 enrolled in the Safe Passage Study conducted by the Prenatal Alcohol and SIDS and Stillbirth (PASS) Network, a multi-center study investigating the role of prenatal exposure in risk for sudden infant death syndrome (SIDS), stillbirth, and fetal alcohol spectrum disorders. Eligibility criteria for the Safe Passage study included the ability to provide informed consent in English or Afrikaans, 16 years of age or older at the time of consent, and a gestational age between 6 weeks and 40 weeks at the time of consent based on estimated delivery date[27]. Informed consent was obtained for the Safe Passage study and from a parent or legal guardian of each participant for developmental follow-up assessments. Ethical approval was obtained for both time points from the Health Research Ethics Committee of Stellenbosch University and the New York State Psychiatric Institute.

Maternal Assessments.

Maternal-infant chart abstraction, demographic, and socioeconomic measures. Maternal-infant medical charts were abstracted to obtain maternal age at delivery, gestational age at birth, mode of delivery, and the infant's biological sex. Measures to collect prenatal alcohol, tobacco, and recreational drug exposure have been previously described[27 28]. Through study specific case report forms, participants indicated demographic and socioeconomic information including race, maternal educational attainment, household crowding (persons per room in household), access to running water inside the house, prenatal care during pregnancy, and marital status.

Self-reported depression and anxiety measures. Information regarding maternal mental health during pregnancy was obtained at 20 – 24 weeks' gestation. Depressive symptoms were

For peer review only - http://bmjopen.bmj.com/site/about/guidelines.xhtml

measured using the Edinburgh Postnatal Depression Scale (EPDS), a depression screening tool developed to specifically assess depressive symptoms in perinatal women where higher scores indicate more severe depressive symptoms[29 30]. The EPDS is widely used and has been validated in English and Afrikaans in South Africa[29 31]. Prior studies have used a cut-off score of ≥12 or ≥ 13 to be indicative of major depression within perinatal women living in South Africa[29 31]. Maternal anxiety symptoms were measured using the State-Trait Anxiety Inventory (STAI)[32], an anxiety screening tool to distinguish anxiety symptoms from depressive symptoms which has also been validated in both languages[33]. The STAI has two subscales, state anxiety which reflects the participant's current state of anxiety when completing the questionnaires and trait anxiety, which is thought to be consistent across time and reflect personality traits. In HICs, the STAI has a cut-off score of ≥ 40 on both the state anxiety and trait anxiety subscales to indicate a threshold for clinical levels of anxiety. Based on these prior studies, we used a cutoff of ≥ 13 to indicate maternal depression, a cutoff of > 40 on the STAI-state subscale to indicate state anxiety, and a cutoff of > 40 on the STAI-trait subscale to indicate trait anxiety.

Toddler Developmental Assessments.

Bayley Scales of Infant Development III Screening Test. The Bayley Scales of Infant Development III (BSID-III screening test) were designed as a rapid assessment of cognitive, language, and motor functioning in infants and young children in order to determine if a child's development is within normal limits and identify risk for developmental delay. The BSID-III screening test has high test-retest reliability: Cognitive (0.85), Receptive Language (0.88), Expressive Language (0.88), Fine Motor (0.82), and Gross Motor (0.86)[34]. Although the BSID-III screening test does not identify degree of impairment, the cut-off points indicate whether a

For peer review only - http://bmjopen.bmj.com/site/about/guidelines.xhtml

child shows competence in age-appropriate tasks, evidence of emerging age-appropriate skills, and evidence of being at risk for developmental delay. The BSID has been validated and widely used throughout South Africa[35 36].

Brief Infant Toddler Social Emotional Assessment (BITSEA). The Brief Infant-Toddler Social and Emotional Assessment (BITSEA) is a 42-item parental report measure of social-emotional development, behavioral problems, and delays in competence[37]. Domains assessed within the BITSEA include: externalizing (activity/impulsivity, aggression/defiance, peer aggression), internalizing (depression/withdrawal, anxiety, separation distress, inhibition to novelty), dysregulation (sleep, negative emotionality, eating, sensory sensitivity), and competence (compliance, attention, imitation/play, mastery motivation, empathy, and pro-social peer relations)[37]. Findings from the BITSEA validation study provide preliminary support for the BITSEA as a reliable and valid brief screener for infant-toddler social-emotional and behavioral problems in addition to delays in competence[38]. When used in socioeconomically and ethnically diverse community-based populations, the BITSEA demonstrated excellent test-retest reliability and good inter-rater agreement between parents[37].

Statistical Analyses. Using multiple linear regression models, we estimated independent and joint effects of maternal depression and state and trait anxiety on social-emotional problem, social emotional competence, and cognitive development scores. Two, separate four-level categorical prenatal maternal mental health variables were created to assess the impact of prenatal maternal depression, trait anxiety, and state anxiety. We created a prenatal maternal depression and trait anxiety variable with four categories: (1) *No Prenatal Depression or Trait Anxiety* (n=199; 33.17%, Reference Category), (2) *Prenatal Depression Only* (106; 17.67%), (3)

For peer review only - http://bmjopen.bmj.com/site/about/guidelines.xhtml

Prenatal Trait Anxiety Only (n=68; 11.33%) and (4) *Prenatal Maternal Depression and Trait Anxiety* (n=227; 37.83%) (Table 1). In separate models we additionally examined the independent and joint effects of prenatal maternal depression and state anxiety. We created a prenatal maternal depression and state anxiety variable with four categories: (1) *No Prenatal Depression or State Anxiety* (n=248; 41.33%; Reference Category), (2) *Prenatal Depression Only* (n=237; 39.50%), (3) *Prenatal State Anxiety Only* (n=19; 3.17%) and (4) *Prenatal Maternal Depression and State Anxiety* (n=96; 16%) (Table 1). For each regression model, either *No Prenatal Maternal Depression or Trait Anxiety* or *No Prenatal Maternal Depression and State Anxiety* was set as the reference category. Minimally adjusted models included sex, gestational age at birth, and age at follow up as covariates. Fully adjusted models additionally controlled for prenatal maternal alcohol use, prenatal maternal tobacco use, maternal employment status at delivery, maternal educational attainment at delivery, parity, and the household crowding index. We used missing indicator methods and median imputation to account for missing categorial and continuous covariate data, respectively (described in Table 1). All analyses were performed in SAS software version 9.4 (SAS Institute, Cary NC).

For peer review only

Results

Maternal and Child Demographic Characteristics. The participants included in the present analysis consisted of mothers and their infant born between April 2014 and August 2015 from the Western Cape Province of South Africa who participated in a follow-up study to examine social-emotional development and cognitive development at approximately three years of age. A total of n=18 mother-infant dyads were excluded due to missing maternal prenatal mental health data. The final sample consisted of 600 maternal-infant dyads (274 females; gestational age at birth: 38.89 weeks ± 2.03) (Table 1).

Child Social-Emotional Development. Based on the BITSEA problem scale percentile rank score of 26 or higher, 50% of children (310/614) were classified as having a "possible problem". Based on the BITSEA competence scale percentile rank score of 15 of lower, 5% (34/614) of children were classified in the "possible deficit/delay range" (Table 2). There were no significant sex differences in social-emotional problems on the BITSEA, however girls had significantly higher social-emotional competence compared to boys (mean difference: 0.38, CI: 0.05, 0.71, p = 0.03)

Association between Prenatal Maternal Depression, Trait Anxiety, and Child Social-Emotional Development. Compared to children born to mothers with no prenatal depression or trait anxiety, children born to mothers with prenatal depression and trait anxiety had the greatest increase in social-emotional problems (mean difference: 4.66; 95% CI 3.43, 5.90), followed by prenatal maternal trait anxiety only (mean difference: 3.87; 95% CI 2.07, 5.66), and finally prenatal maternal depression only (mean difference: 2.76; 95% CI 1.23, 4.29) in minimally adjusted models. These associations remained significant in fully adjusted models where similarly the greatest increase in social-emotional problems was in the comorbid prenatal

For peer review only - http://bmjopen.bmj.com/site/about/guidelines.xhtml

maternal depression and trait anxiety group (mean difference: 4.33; 95% CI 2.90, 5.67), followed by prenatal maternal trait anxiety only (mean difference: 3.23; 95% CI 1.19, 5.27), with the smallest mean difference for prenatal maternal depression only group (mean difference: 2.64; 95% CI 1.02, 4.27) as compared to the no prenatal depression or trait anxiety group (Figure 1). Additional significant predictors in the multivariate models were parity of 3 or greater which was associated with a reduction in social-emotional problems (mean difference: -3.02, 95% CI -4.63, -1.40) and low continuous smoking during pregnancy which was associated with an increase in social-emotional problems (mean difference: 1.39, 95% CI 0.039, 2.74). There were no significant associations between prenatal maternal depression, trait anxiety, and child social-emotional competence.

Association between Prenatal Maternal Depression, State Anxiety, and Child Social-Emotional Development. Compared to children born to mothers with no prenatal depression or state anxiety, children born to mothers with comorbid prenatal depression and state anxiety had the greatest increase in social-emotional problems (mean difference: 4.29; 95% CI 2.73, 5.84). Children born to mothers with prenatal depression only also had increased social-emotional problems compared to mothers with no prenatal depression or state anxiety (mean difference: 2.71: 95% CI 1.51, 3.88). These associations remained significant in fully adjusted models (prenatal depression and state anxiety: 3.90 mean increase (95% CI 2.19, 5.60); prenatal depression only: 2.58 mean increase (95% CI 1.34, 3.82)) (Figure 1). Additional significant predictors in the multivariate models were parity of 3 or greater which was associated with a mean reduction in social-emotional problems (mean difference: -3.22, 95% CI -4.86, -1.58), low continuous smoking during pregnancy which was associated with a mean increase in social-emotional problems (mean difference: 1.44; 95% CI 0.075, 2.81), and finally less than a high

For peer review only - http://bmjopen.bmj.com/site/about/guidelines.xhtml

school education (some primary school only) which was associated with a mean increase in social-emotional problems (mean difference: 3.47; 95% CI 0.248, 6.70). However, there was no significant mean difference in social-emotional problems on the BITSEA in children born to mothers with prenatal state anxiety only. There were also no significant associations between prenatal maternal depression, state anxiety, and social-emotional competence.

Child Cognitive, Language, and Motor Development. Based on normative cognitive cutoff scores defined by the BSID-III, 4% of children (24/615) were classified as at-risk, 73% of children were classified as emerging (448/615), and 23% of children were classified as competent (143/615) (Table 2). Risk classification percentages were similar across all subdomains, expressive language: 5% , receptive language: 4%, gross motor: 3%, and fine motor: 3%. There were no significant sex differences in child cognitive scores.

Association between Prenatal Maternal Depression, Trait Anxiety, and Child Cognitive, Language, and Motor Development. Compared to children born to mothers with no prenatal depression or trait anxiety, children born to mothers with comorbid prenatal depression and trait anxiety had decreased mean cognitive scores on the BSID-III (mean difference: -1.04; 95% CI -1.99, -0.08). Results remained significant in the fully adjusted model (mean difference; -1.11; 95% CI -2.13, -0.09) (Figure 2). Low continuous prenatal maternal alcohol use was also associated with a mean reduction in cognitive scores (mean difference: -1.30; 95% CI -2.36, -0.24).

Association between Prenatal Maternal Depression, State Anxiety, and Child Cognitive, Language, and Motor Development. Compared to children born to mothers with neither prenatal depression nor trait anxiety, there was no significant reduction in cognitive scores for children

For peer review only - http://bmjopen.bmj.com/site/about/guidelines.xhtml

born to mothers with prenatal depression only, prenatal trait anxiety only, or combined prenatal depression and trait anxiety in either the minimally or fully adjusted models.

Discussion

In summary, we found the greatest increase in child social-emotional problems in children born to women with comorbid prenatal depression and trait anxiety compared to women with no prenatal depression or trait anxiety, prenatal depression alone, or prenatal trait anxiety alone. We additionally found a significant effect of comorbid prenatal maternal depression and state anxiety on increased child social-emotional problems; however, we found no effect of prenatal maternal state anxiety in the absence of prenatal maternal depression on child social-emotional problems. Finally, we reported children born to mothers with prenatal maternal depression and trait anxiety had lower cognitive scores on the BSID-III, but we did not find an association between prenatal maternal depression alone on cognitive outcomes at 3-years of age.

Our finding linking prenatal maternal depression and anxiety to social-emotional risk parallels two recent South African studies linking increased prenatal maternal stressors to increased behavioral problems in children with an odds ratio of 2.52, but not before 4 years of age[25] and increased perinatal depression to aggressive behaviors at 60 months of age[20]. Our findings are also largely consistent with a recent meta-analysis of studies predominately conducted in HICs, which found prenatal maternal depression and anxiety were associated with increased social-emotional problems in offspring with larger effect sizes for prenatal maternal depression (OR 1.79; 95% CI 1.61 - 1.99) compared to prenatal maternal anxiety (OR 1.50, 95% CI, 1.36 - 1.64)[6]. We found the greatest increase in child social-emotional problems in children born to women with comorbid prenatal depression and trait anxiety and no effect of prenatal maternal state anxiety in the absence of prenatal maternal depression on child social-emotional

For peer review only - http://bmjopen.bmj.com/site/about/guidelines.xhtml

problems. Our results are suggestive that chronic anxiety measured via trait anxiety may be more predictive of child neurobehavioral outcomes than a single measurement of concurrent anxiety during pregnancy.

A prior meta-analysis[6] also found stronger effects of prenatal maternal depression and anxiety on child outcomes when sociodemographic risk factors such as low-income, lower levels of parental education, or single family households, were highest[6]. Similarly, we found that lower levels of maternal education, low continuous tobacco use during pregnancy, and low continuous alcohol use during pregnancy were associated with increased social-emotional problems in children at three years of age. Intriguingly, maternal parity of 3 or greater was protective and associated with a mean reduction in social-emotional problems which may be due to reduced depression and anxiety levels in women with higher parity, changes in the perception of their child's behavior due to having multiple children, or additional social opportunities during sibling interactions[39 40].

The Drakenstein Child Health Study based in the Western Cape found approximately 50% of the overall sample (369/731) were categorized as having cognitive delay at two years of age based on cutoffs defined by United States normative data[26]. Better cognitive outcomes were associated with higher maternal education, older child age, a primigravid mother, and higher socioeconomic status whereas prenatal maternal depression was associated with a 1.03 SD (95% CI -1.94 to -0.12) reduction in cognitive scores on the Bayley Scales of Infant and Toddler Development at two years of age[26]. Similar to the Drakenstein Child Health Study[26], we found children born to mothers with prenatal maternal depression and trait anxiety had lower cognitive scores on the BSID-III compared to the no prenatal maternal depression or trait anxiety group. However, in contrast we did not find an association between prenatal maternal depression alone

For peer review only - http://bmjopen.bmj.com/site/about/guidelines.xhtml

on cognitive outcomes at 3-years of age. More recently, a large home-visiting intervention study based in Cape Town examined the effect of prenatal maternal depression only, postnatal maternal depression only (birth – 60 months), or recurrent pre- and postnatal maternal depression. This study also accounted for several other risk factors including intimate partner violence, HIV status, and alcohol use on child social behaviors, language skills, and cognitive development. No associations were found between maternal depression at any time point with children's language or cognitive development at 36 or 60 months of age[20]. However, children of never depressed mothers had lower aggressive behaviors on the Child Behavior Checklist at 60 months of age than children of mothers with postnatal depression only or perinatal depression[20].

There are several biological mechanisms which can explain prior studies and our current findings linking prenatal maternal depression and anxiety with child social-emotional behaviors and cognitive development including increased prenatal maternal inflammation and/or increased cortisol production[41-50]. Pregnancy is associated with increased inflammatory processes and increased placental cortisol production with reduced maternal hypothalamic–pituitary–adrenal (HPA) axis sensitivity to stress[41 42]. However, prenatal maternal depression may upregulate inflammatory processes and/or cortisol production. Maternal cortisol can cross the placenta resulting in increased inflammation[41 42] and/or affect the developmental of limbic regions, which are associated with social and emotional processes[51]. In animal models, increased proinflammatory proteins have been associated with widespread changes in perinatal brain development such as with volume reductions in gray and white matter, decreased density of GABAergic neurons, reduced synaptic pruning, and network dysfunction with potential downstream effects on neurobehavioral development[43-50].

For peer review only - http://bmjopen.bmj.com/site/about/guidelines.xhtml

While we report an association of prenatal maternal depression and anxiety with child social-emotional and cognitive development from a prospective cohort study with a fairly large sample of participants, there are several limitations within the current study that are worth noting. Although, prior studies have emphasized the importance of measuring childhood trauma, maternal stress, and social support within communities with several heterogeneous risk factors[3], these measures were not collected as part of the original NIH Safe Passage Study or our recent follow-up study. Therefore, we could not examine their effects or consider maternal social support as a potential moderator of resilient neurodevelopmental outcomes. There is also robust literature examining both postpartum depression (PPD) and the early mother-infant relation in shaping child outcomes. Two prior studies in South Africa demonstrated maternal intrusiveness and coerciveness mediated the association between maternal PPD and early childhood attachment[52 53]. In the current study, we did not collect data postnatally between birth and three years of age. Additionally, we did not collect postnatal information on maternal depression, anxiety, or stress. Therefore, we cannot draw conclusions regarding the combined effect of the pre- and postnatal environment on child neurodevelopmental outcomes. Strengths of the current analyses include our large sample size, evaluation of both cognitive and social-emotional outcomes within the same cohort, finally the detailed prospective collection of both depression and anxiety symptoms during pregnancy[27] in addition to other potential confounders.

Our results suggest comorbid prenatal maternal depression and chronic anxiety have a greater impact on child social-emotional and cognitive development than either condition alone or than comorbid prenatal maternal depression and transitory anxiety during pregnancy. These findings are supported by a robust literature within the DOHaD framework linking perturbations in the gestational environment to later neurodevelopmental or psychiatric sequelae. Our results

For peer review only

also lend support for future intervention studies aimed at perinatal mental health interventions targeting maternal depressive and anxiety symptoms to improve long-term child social-emotional and cognitive developmental outcomes.

For peer review only

For peer review only - http://bmjopen.bmj.com/site/about/guidelines.xhtml

Table 1 - Sociodemographic characteristics		
	Mean ± SD or N (%)	N and Percent Missing (%)
**Maternal Characteristics**		
Maternal Age (years)	25.26 ± 5.91	0 (0%)
Maternal Body Mass Index (BMI)		0 (0%)
BMI <18.5 (kg/m2)	62 (10.33%)	
BMI 18.5-25 (kg/m2)	282 (47%)	
BMI 25-30 (kg/m2)	132 (22%)	
BMI >30 (kg/m2)	124 (20.67%)	
Parity		0 (0%)
Parity <1	218 (36.33%)	
Parity =1	196 (32.67%)	
Parity =2	103 (17.17%)	
Parity >=3	83 (13.83%)	
Antenatal care visits		0 (0%)
antenatal care visit <3	49 (8.17%)	
antenatal care visit 3-6	389 (64.83%)	
antenatal care visit >6	162 (27%)	
Cesarean Section	106 (17.70%)	1 (0.1%)
Education		0 (0%)
Some primary school	43 (7.167%)	
Some high school	405 (67.57%)	
Completed high school	119 (19.83%)	
Beyond high school	33 (5.5%)	
Married	290 (48.33%)	0 (0%)
Employed	172 (28.67%)	0 (0%)
Adjusted Household Crowding	1.56 ± 0.72	2 (0.33%)
Depression (Edinburgh >=13)	333 (55%)	0 (0%)
Anxiety (State-Trait Anxiety Inventory >=40)	115 (19.17%)	0 (0%)
Maternal Prenatal Alcohol Use		0 (0%)
Non drinking	245 (46.93%)	
Moderate-high continuous drinking	122 (23.37%)	
Low continuous drinking	26 (4.98%)	
Quit early drinking	129 (24.71%)	
Maternal Prenatal Tobacco Use		2 (0.33%)
Non smoking	227 (37.97%)	

For peer review only

Moderate-high continuous smoking	132 (22.07%)	
Low continuous smoking	222 (37.12%)	
Quit early smoking	17 (2.84%)	
Raw Maternal Edinburgh Score	12.99 ± 5.73	0 (0%)
Raw Maternal State Anxiety Score	31.23 ± 10.24	0 (0%)
Raw Maternal Trait Anxiety Score	40.63 ± 10.63	0 (0%)
Depression - Trait Anxiety Groups		0 (0%)
No depression or trait anxiety	199 (33.17%)	
Depression alone	106 (17.67%)	
Trait anxiety alone	68 (11.33%)	
Depression and trait anxiety	227 (37.83%)	
Depression - State Anxiety Groups		0 (0%)
No depression or state anxiety	248 (41.33%)	
Depression alone	237 (39.50%)	
State anxiety alone	19 (3.17%)	
Depression and state anxiety	96 (16%)	
**Infant Characteristics**		
Infant Sex		0 (0%)
Male	326 (54.33%)	
Female	274 (45.67%)	
Gestational age at birth (weeks)	38.89 ± 2.03	0 (0%)
Infant Birth weight (grams)	2980.65 ± 564.65	2 (0.33%)
Follow-up age (months)	38.29 ± 2.96	0 (0%)
Adjusted follow-up age (months)	38.14 ± 0.016	0 (0%)

**Table 2. Neurodevelopmental Outcome Raw Scores and At-risk Groups**	
Raw Mullen Scores	
Gross motor	25.47 ± 4.34
Fine motor	23.55 ± 4.08
Problem solving	13.40 ± 6.77
Receptive language	21.69 ± 5.10
Expressive language	21.27 ± 3.78
At-Risk Categories	
Gross motor	21 (3.5%)
Fine motor	18 (3%)
Cognitive	24 (4.0%)
Receptive language	33 (5.50%)
Expressive language	25 (4.17%)

For peer review only - http://bmjopen.bmj.com/site/about/guidelines.xhtml

Brief Infant Toddler Social-Emotional Assessment (BITSEA)		
	Social Emotional Problem	13.4 ± 6.77
	Competence	19.55 ± 2.07
At-Risk Categories		
	Social Emotional Problem	310 (50%)
	Competence	34 (5%)

For peer review only

References

1. O'Donnell KJ, Meaney MJ. Fetal Origins of Mental Health: The Developmental Origins of Health and Disease Hypothesis. *Am J Psychiatry* 2017;174(4):319-28. doi: 10.1176/appi.ajp.2016.16020138 [published Online First: 2016/11/15]
2. Hofer MA. The Roots of Human Behavior: An Introduction to the Psychobiology of Early Development. San Francisco, CA: W. H. Freeman & Company 1981.
3. Stein DJ, Koen N, Donald KA, et al. Investigating the psychosocial determinants of child health in Africa: The Drakenstein Child Health Study. *J Neurosci Methods* 2015;252:27-35. doi: 10.1016/j.jneumeth.2015.03.016 [published Online First: 2015/03/24]
4. Herba CM, Glover V, Ramchandani PG, et al. Maternal depression and mental health in early childhood: an examination of underlying mechanisms in low-income and middle-income countries. *Lancet Psychiatry* 2016;3(10):983-92. doi: 10.1016/S2215-0366(16)30148-1 [published Online First: 2016/09/22]
5. Monk C, Lugo-Candelas C, Trumpff C. Prenatal Developmental Origins of Future Psychopathology: Mechanisms and Pathways. *Annu Rev Clin Psychol* 2019;15:317-44. doi: 10.1146/annurev-clinpsy-050718-095539 [published Online First: 2019/02/24]
6. Madigan S, Oatley H, Racine N, et al. A Meta-Analysis of Maternal Prenatal Depression and Anxiety on Child Socioemotional Development. *J Am Acad Child Adolesc Psychiatry* 2018;57(9):645-57 e8. doi: 10.1016/j.jaac.2018.06.012
7. King S, Laplante DP. The effects of prenatal maternal stress on children's cognitive development: Project Ice Storm. *Stress* 2005;8(1):35-45. doi: 10.1080/10253890500108391
8. Huizink AC, Robles de Medina PG, Mulder EJ, et al. Stress during pregnancy is associated with developmental outcome in infancy. *J Child Psychol Psychiatry* 2003;44(6):810-8. doi: 10.1111/1469-7610.00166 [published Online First: 2003/09/10]
9. Leis JA, Heron J, Stuart EA, et al. Associations between maternal mental health and child emotional and behavioral problems: does prenatal mental health matter? *J Abnorm Child Psychol* 2014;42(1):161-71. doi: 10.1007/s10802-013-9766-4
10. O'Connor TG, Heron J, Golding J, et al. Maternal antenatal anxiety and children's behavioural/emotional problems at 4 years. Report from the Avon Longitudinal Study of Parents and Children. *Br J Psychiatry* 2002;180:502-8.
11. O'Donnell KJ, Glover V, Barker ED, et al. The persisting effect of maternal mood in pregnancy on childhood psychopathology. *Dev Psychopathol* 2014;26(2):393-403. doi: 10.1017/S0954579414000029

12. Loomans EM, van der Stelt O, van Eijsden M, et al. Antenatal maternal anxiety is associated with problem behaviour at age five. *Early Hum Dev* 2011;87(8):565-70. doi: 10.1016/j.earlhumdev.2011.04.014
13. Rice F, Harold GT, Boivin J, et al. The links between prenatal stress and offspring development and psychopathology: disentangling environmental and inherited influences. *Psychol Med* 2010;40(2):335-45. doi: 10.1017/S0033291709005911
14. Ronald A, Pennell CE, Whitehouse AJ. Prenatal Maternal Stress Associated with ADHD and Autistic Traits in early Childhood. *Front Psychol* 2010;1:223. doi: 10.3389/fpsyg.2010.00223 [published Online First: 2010/01/01]
15. Lereya ST, Wolke D. Prenatal family adversity and maternal mental health and vulnerability to peer victimisation at school. *J Child Psychol Psychiatry* 2013;54(6):644-52. doi: 10.1111/jcpp.12012
16. Rogers A, Obst S, Teague SJ, et al. Association Between Maternal Perinatal Depression and Anxiety and Child and Adolescent Development: A Meta-analysis. *JAMA Pediatr* 2020;174(11):1082-92. doi: 10.1001/jamapediatrics.2020.2910 [published Online First: 2020/09/15]
17. Van den Bergh BRH, van den Heuvel MI, Lahti M, et al. Prenatal developmental origins of behavior and mental health: The influence of maternal stress in pregnancy. *Neurosci Biobehav Rev* 2017 doi: 10.1016/j.neubiorev.2017.07.003 [published Online First: 2017/08/02]
18. Eshetu EB, Woldesenbet SA. Are there particular social determinants of health for the world's poorest countries? *African Health Sciences* 2011;11(1):108 - 15.
19. Davies T, Rahman A, Lund C. Psychotherapy for perinatal mental disorders in low- and middle-income countries. In Global Mental Health in Practice, Global Mental Health and Psychotherapy: Academic Press, 2019:301 - 19.
20. Gordon S, Rotheram-Fuller E, Rezvan P, et al. Maternal depressed mood and child development over the first five years of life in South Africa. *J Affect Disord* 2021;294:346-56. doi: 10.1016/j.jad.2021.07.027 [published Online First: 2021/07/28]
21. Heyningen TV, Myer L, Onah M, et al. Antenatal depression and adversity in urban South Africa. *J Affect Disord* 2016;203:121-29. doi: 10.1016/j.jad.2016.05.052 [published Online First: 2016/06/11]
22. Redinger S, Norris SA, Pearson RM, et al. First trimester antenatal depression and anxiety: prevalence and associated factors in an urban population in Soweto, South Africa. *J Dev Orig Health Dis* 2018;9(1):30-40. doi: 10.1017/S204017441700071X [published Online First: 2017/09/08]
23. Redinger S, Pearson RM, Houle B, et al. Antenatal depression and anxiety across pregnancy in urban South Africa. *J Affect Disord* 2020;277:296-305. doi: 10.1016/j.jad.2020.08.010 [published Online First: 2020/08/29]
24. Drysdale RE, Slemming W, Makusha T, et al. Father involvement, maternal depression and child nutritional outcomes in Soweto, South Africa. *Matern Child Nutr* 2021;17 Suppl 1:e13177. doi: 10.1111/mcn.13177 [published Online First: 2021/07/10]
25. Ramchandani PG, Richter LM, Norris SA, et al. Maternal prenatal stress and later child behavioral problems in an urban South African setting. *J Am Acad Child Adolesc Psychiatry* 2010;49(3):239-47. [published Online First: 2010/04/23]
26. Donald KA, Wedderburn CJ, Barnett W, et al. Risk and protective factors for child development: An observational South African birth cohort. *PLoS Med*

For peer review only - http://bmjopen.bmj.com/site/about/guidelines.xhtml

2019;16(9):e1002920. doi: 10.1371/journal.pmed.1002920 [published Online First: 2019/09/29]

27. Dukes KA, Burd L, Elliott AJ, et al. The Safe Passage Study: Design, Methods, Recruitment, and Follow-Up Approach. *Paediatr Perinat Ep* 2014;28(5):455-65. doi: 10.1111/ppe.12136
28. Dukes K, Tripp T, Petersen J, et al. A modified Timeline Followback assessment to capture alcohol exposure in pregnant women: Application in the Safe Passage Study. *Alcohol* 2017;62:17-27. doi: 10.1016/j.alcohol.2017.02.174
29. Lawrie TA, Hofmeyr GJ, de Jager M, et al. Validation of the Edinburgh Postnatal Depression Scale on a cohort of South African women. *S Afr Med J* 1998;88(10):1340-4. [published Online First: 1998/11/10]
30. Alvarado R, Jadresic E, Guajardo V, et al. First validation of a Spanish-translated version of the Edinburgh postnatal depression scale (EPDS) for use in pregnant women. A Chilean study. *Arch Womens Ment Health* 2015;18(4):607-12. doi: 10.1007/s00737-014-0466-z [published Online First: 2014/10/11]
31. Hartley M, Tomlinson M, Greco E, et al. Depressed mood in pregnancy: prevalence and correlates in two Cape Town peri-urban settlements. *Reprod Health* 2011;8:9. doi: 10.1186/1742-4755-8-9 [published Online First: 2011/05/04]
32. Spielberger CD. Manual for the State-Trait Anxiety Inventory: STAI. Palo Alto, CA: Consulting Psychologists Press, 1983.
33. Pretorius TB, Norman AM. Psychometric Data on the Statistics Anxiety Scale for a Sample of South-African Students. *Educational and Psychological Measurement* 1992;52(4):933-37. doi: Doi 10.1177/0013164492052004015
34. Bayley N. Bayley scales of infant and toddler development: Bayley-III San Antonio, Texas, USA: Harcourt Assessment, Psych. Corporation. 2006.
35. Rademeyer V, Jacklin L. A study to evaluate the performance of black South African urban infants on the Bayley Scales of Infant Development III. *South African Journal of Child Health* 2013;7(2):54-59.
36. Ballot DE, Ramdin T, Rakotsoane D, et al. Use of the Bayley Scales of Infant and Toddler Development, Third Edition, to Assess Developmental Outcome in Infants and Young Children in an Urban Setting in South Africa. *Int Sch Res Notices* 2017;2017:1631760. doi: 10.1155/2017/1631760 [published Online First: 2017/08/25]
37. Gowan B, Carter MJ, Carter AS. ITSEA/BITSEA: Infant-Toddler and Brief Infant-Toddler Social and Emotional Assessment. *The Psychological Corporation* 2006
38. Kruizinga I, Visser JC, van Batenburg-Eddes T, et al. Screening for autism spectrum disorders with the brief infant-toddler social and emotional assessment. *PLoS One* 2014;9(5):e97630. doi: 10.1371/journal.pone.0097630 [published Online First: 2014/05/24]
39. Sang S, Nelson, JA. The effect of siblings on children's social skills and perspective taking. *Inf Child Dev* 201, 26:e2023. doi: 10.1002/icd.2023.
40. Downey DB CD, Yucel D. Number of siblings and social skills revisited among american fifth graders. *Journal of Family Issues* 2015, 36(2):273-96. doi: 10.1177/0192513X13507569
41. Ghassabian A, Albert PS, Hornig M, et al. Gestational cytokine concentrations and neurocognitive development at 7 years. *Transl Psychiat* 2018;8 doi: ARTN 64

10.1038/s41398-018-0112-z

For peer review only - http://bmjopen.bmj.com/site/about/guidelines.xhtml

42. Ruiz RJ, Avant KC. Effects of maternal prenatal stress on infant outcomes - A synthesis of the literature. *Adv Nurs Sci* 2005;28(4):345-55. doi: Doi 10.1097/00012272-200510000-00006
43. Short SJ, Lubach GR, Karasin AI, et al. Maternal influenza infection during pregnancy impacts postnatal brain development in the rhesus monkey. *Biol Psychiatry* 2010;67(10):965-73. doi: 10.1016/j.biopsych.2009.11.026 [published Online First: 2010/01/19]
44. Paolicelli RC, Bolasco G, Pagani F, et al. Synaptic pruning by microglia is necessary for normal brain development. *Science* 2011;333(6048):1456-8. doi: 10.1126/science.1202529 [published Online First: 2011/07/23]
45. Bergdolt L, Dunaevsky A. Brain changes in a maternal immune activation model of neurodevelopmental brain disorders. *Prog Neurobiol* 2019;175:1-19. doi: 10.1016/j.pneurobio.2018.12.002 [published Online First: 2018/12/28]
46. Meyer U. Neurodevelopmental Resilience and Susceptibility to Maternal Immune Activation. *Trends Neurosci* 2019;42(11):793-806. doi: 10.1016/j.tins.2019.08.001 [published Online First: 2019/09/09]
47. Patterson PH. Maternal infection: window on neuroimmune interactions in fetal brain development and mental illness. *Curr Opin Neurobiol* 2002;12(1):115-8. doi: 10.1016/s0959-4388(02)00299-4 [published Online First: 2002/02/28]
48. Bland ST, Beckley JT, Young S, et al. Enduring consequences of early-life infection on glial and neural cell genesis within cognitive regions of the brain. *Brain Behav Immun* 2010;24(3):329-38. doi: 10.1016/j.bbi.2009.09.012 [published Online First: 2009/09/29]
49. Money KM, Barke TL, Serezani A, et al. Gestational diabetes exacerbates maternal immune activation effects in the developing brain. *Mol Psychiatry* 2018;23(9):1920-28. doi: 10.1038/mp.2017.191 [published Online First: 2017/09/28]
50. Bilbo SD, Schwarz JM. Early-life programming of later-life brain and behavior: a critical role for the immune system. *Front Behav Neurosci* 2009;3:14. doi: 10.3389/neuro.08.014.2009 [published Online First: 2009/09/10]
51. Buss C, Davis EP, Shahbaba B, et al. Maternal cortisol over the course of pregnancy and subsequent child amygdala and hippocampus volumes and affective problems. *P Natl Acad Sci USA* 2012;109(20):E1312-E19. doi: 10.1073/pnas.1201295109
52. Cooper PJ, Tomlinson M, Swartz L, et al. Post-partum depression and the mother-infant relationship in a South African peri-urban settlement. *Br J Psychiatry* 1999;175:554-8. doi: 10.1192/bjp.175.6.554 [published Online First: 2000/05/02]
53. Tomlinson M, Cooper P, Murray L. The mother-infant relationship and infant attachment in a South African peri-urban settlement. *Child Dev* 2005;76(5):1044-54. doi: 10.1111/j.1467-8624.2005.00896.x [published Online First: 2005/09/10]

For peer review only

Contributors: WPF and HJO acquired funding for and designed the work. LCS, AS, NHB, ML, YR, PES, HJO, and WPF conceptualized the work. MP, PES, CDP, and HJO acquired the data. LCS, NHB, and WPF led in data collection oversight and quality control. AS, LCS, and YR analyzed the data. All authors contributed to the interpretation of data and drafting the work and revising it critically for important intellectual content. All authors approved the final manuscript and agree to be accountable for all aspects of the work.

Acknowledgements: We would like to acknowledge Lucy Brink, J. David Nugent, and Daianna Rodriguez for their database and administrative support. We would also like to thank all of the families who participated in this research study for making this work possible.

Ethics statements

Patient consent for publication

Not applicable.

Ethics approval

Ethics approval was received from the Institutional Review Boards and ethics review committees at Stellenbosch University (N16-08-101 and N06-10-210) and the New York State Psychiatric Institute (5338). All participants provided informed written consent at both time points (prenatal and postnatal) before inclusion in the study.

For peer review only

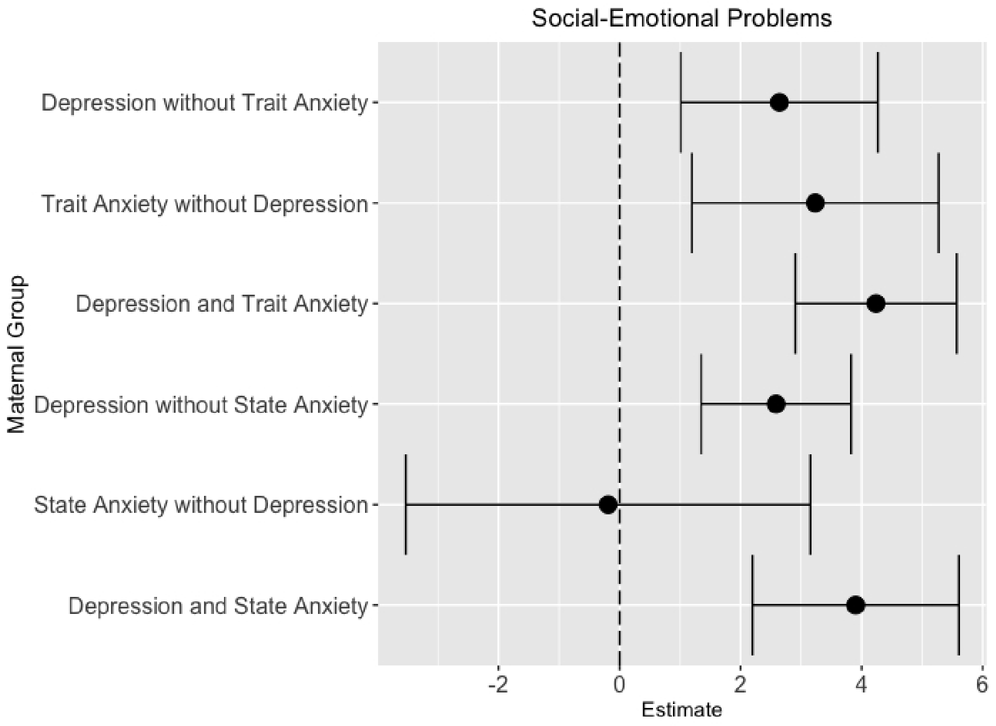

Figure 1. Association between prenatal maternal depression, prenatal maternal anxiety, and child social-emotional problems measured by the BITSEA. Each line plot depicts the mean difference and their confidence interval for child social-emotional problems (x-axis) for each prenatal maternal mental health group (y-axis). Either no prenatal maternal depression and state anxiety or no prenatal maternal depression and trait anxiety were the reference groups. Models were adjusted for sex, gestational age at birth, age at follow-up, prenatal maternal alcohol use, prenatal maternal tobacco use, maternal employment status at delivery, maternal educational attainment at delivery, parity, and the household crowding index.

247x181mm (72 x 72 DPI)

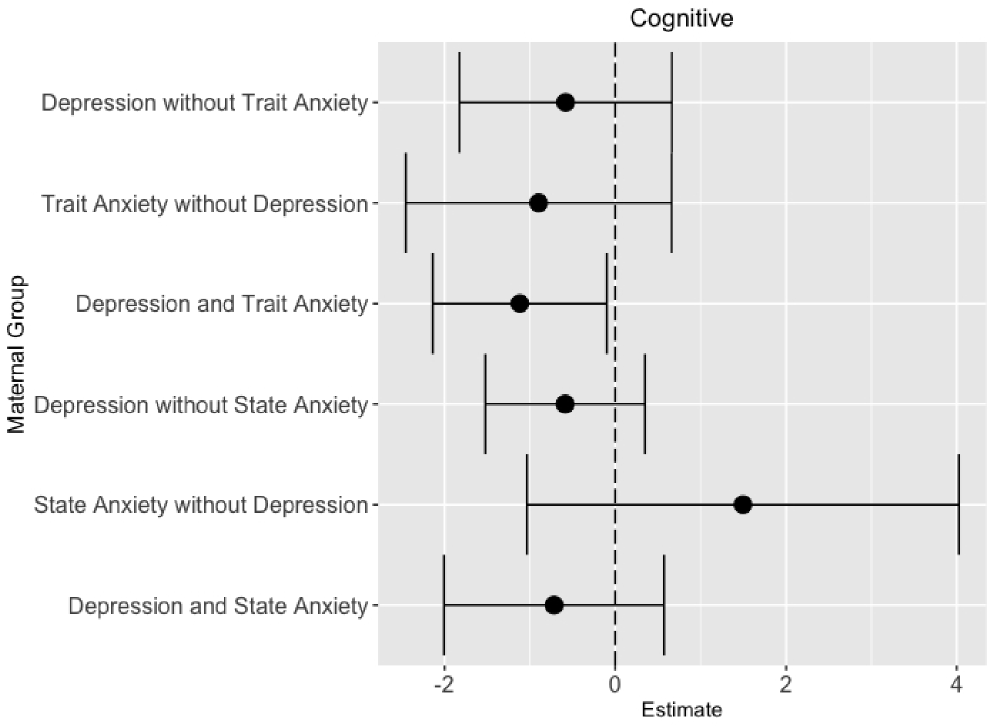

Figure 2. . Association between prenatal maternal depression, prenatal maternal anxiety, and child cognitive development measured by the BSID-III Screening Test. Each line plot depicts the mean difference and their confidence interval for child cognitive development (x-axis) for each prenatal maternal mental health group (y-axis). Either no prenatal maternal depression and state anxiety or no prenatal maternal depression and trait anxiety were the reference groups. Models were adjusted for sex, gestational age at birth, age at follow-up, prenatal maternal alcohol use, prenatal maternal tobacco use, maternal employment status at delivery, maternal educational attainment at delivery, parity, and the household crowding index.

247x181mm (72 x 72 DPI)

STROBE Statement—Checklist of items that should be included in reports of ***cohort studies***

	Item No	Recommendation	Page No
**Title and abstract**	1	(*a*) Indicate the study's design with a commonly used term in the title or the abstract	2
		(*b*) Provide in the abstract an informative and balanced summary of what was done and what was found	3, 4
**Introduction**			
Background/rationale	2	Explain the scientific background and rationale for the investigation being reported	5, 6
Objectives	3	State specific objectives, including any prespecified hypotheses	6
**Methods**			
Study design	4	Present key elements of study design early in the paper	7 - 9
Setting	5	Describe the setting, locations, and relevant dates, including periods of recruitment, exposure, follow-up, and data collection	7 - 9
Participants	6	(*a*) Give the eligibility criteria, and the sources and methods of selection of participants. Describe methods of follow-up	7 - 9
		(*b*) For matched studies, give matching criteria and number of exposed and unexposed	N/A
Variables	7	Clearly define all outcomes, exposures, predictors, potential confounders, and effect modifiers. Give diagnostic criteria, if applicable	9 - 10
Data sources/ measurement	8*	For each variable of interest, give sources of data and details of methods of assessment (measurement). Describe comparability of assessment methods if there is more than one group	7 - 10
Bias	9	Describe any efforts to address potential sources of bias	10
Study size	10	Explain how the study size was arrived at	11
Quantitative variables	11	Explain how quantitative variables were handled in the analyses. If applicable, describe which groupings were chosen and why	9 - 10
Statistical methods	12	(*a*) Describe all statistical methods, including those used to control for confounding	9 - 10
		(*b*) Describe any methods used to examine subgroups and interactions	9 - 10
		(*c*) Explain how missing data were addressed	10
		(*d*) If applicable, explain how loss to follow-up was addressed	10
		(*e*) Describe any sensitivity analyses	N/A
**Results**			
Participants	13*	(a) Report numbers of individuals at each stage of study—eg numbers potentially eligible, examined for eligibility, confirmed eligible, included in the study, completing follow-up, and analysed	11
		(b) Give reasons for non-participation at each stage	11
		(c) Consider use of a flow diagram	
Descriptive data	14*	(a) Give characteristics of study participants (eg demographic, clinical, social) and information on exposures and potential confounders	9, 11 - 13
		(b) Indicate number of participants with missing data for each variable of interest	10
		(c) Summarise follow-up time (eg, average and total amount)	
Outcome data	15*	Report numbers of outcome events or summary measures over time	11

For peer review only - http://bmjopen.bmj.com/site/about/guidelines.xhtml

Main results	16	(*a*) Give unadjusted estimates and, if applicable, confounder-adjusted estimates and their precision (eg, 95% confidence interval). Make clear which confounders were adjusted for and why they were included	11 - 13
		(*b*) Report category boundaries when continuous variables were categorized	N/A
		(*c*) If relevant, consider translating estimates of relative risk into absolute risk for a meaningful time period	
Other analyses	17	Report other analyses done—eg analyses of subgroups and interactions, and sensitivity analyses	11 - 13
**Discussion**			
Key results	18	Summarise key results with reference to study objectives	14
Limitations	19	Discuss limitations of the study, taking into account sources of potential bias or imprecision. Discuss both direction and magnitude of any potential bias	17
Interpretation	20	Give a cautious overall interpretation of results considering objectives, limitations, multiplicity of analyses, results from similar studies, and other relevant evidence	17
Generalisability	21	Discuss the generalisability (external validity) of the study results	17
**Other information**			
Funding	22	Give the source of funding and the role of the funders for the present study and, if applicable, for the original study on which the present article is based	1

*Give information separately for exposed and unexposed groups.

Note: An Explanation and Elaboration article discusses each checklist item and gives methodological background and published examples of transparent reporting. The STROBE checklist is best used in conjunction with this article (freely available on the Web sites of PLoS Medicine at http://www.plosmedicine.org/, Annals of Internal Medicine at http://www.annals.org/, and Epidemiology at http://www.epidem.com/). Information on the STROBE Initiative is available at http://www.strobe-statement.org.

For peer review only

BMJ Open

Association of Maternal Depression and Anxiety with Toddler Social-Emotional and Cognitive Development in South Africa: A prospective cohort study

Journal:	*BMJ Open*
Manuscript ID	bmjopen-2021-058135.R1
Article Type:	Original research
Date Submitted by the Author:	22-Feb-2022
Complete List of Authors:	Shuffrey, Lauren; Columbia University Irving Medical Center, Psychiatry; New York State Psychiatric Institute, Division of Developmental Neuroscience<br>Sania, Ayesha; Columbia University Irving Medical Center, Psychiatry; New York State Psychiatric Institute, Division of Developmental Neuroscience<br>Brito, Natalie; New York University, Department of Applied Psychology<br>Potter, Mandy; Stellenbosch University Faculty of Medicine and Health Sciences, Obstetrics and Gynaecology<br>Springer, Priscilla; Stellenbosch University, Paediatrics and Child Health<br>Lucchini, Maristella ; Columbia University Irving Medical Center, Psychiatry; New York State Psychiatric Institute, Neuroscience<br>Rayport, Yael; Columbia University Irving Medical Center, Department of Psychiatry; New York State Psychiatric Institute, Neuroscience<br>Du Plessis, Carlie; Stellenbosch University Faculty of Medicine and Health Sciences, Department of Obstetrics and Gynaecology<br>Odendaal, H. J.; Stellenbosch University Faculty of Medicine and Health Sciences, Obstetrics & Gynaecology<br>Fifer, William; Columbia University Irving Medical Center, Psychiatry; New York State Psychiatric Institute, Neuroscience
**Primary Subject Heading**:	Global health
Secondary Subject Heading:	Mental health, Paediatrics
Keywords:	Community child health < PAEDIATRICS, Depression & mood disorders < PSYCHIATRY, Anxiety disorders < PSYCHIATRY, Child & adolescent psychiatry < PSYCHIATRY

SCHOLARONE™
Manuscripts

I, the Submitting Author has the right to grant and does grant on behalf of all authors of the Work (as defined in the below author licence), an exclusive licence and/or a non-exclusive licence for contributions from authors who are: i) UK Crown employees; ii) where BMJ has agreed a CC-BY licence shall apply, and/or iii) in accordance with the terms applicable for US Federal Government officers or employees acting as part of their official duties; on a worldwide, perpetual, irrevocable, royalty-free basis to BMJ Publishing Group Ltd ("BMJ") its licensees and where the relevant Journal is co-owned by BMJ to the co-owners of the Journal, to publish the Work in this journal and any other BMJ products and to exploit all rights, as set out in our licence.

The Submitting Author accepts and understands that any supply made under these terms is made by BMJ to the Submitting Author unless you are acting as an employee on behalf of your employer or a postgraduate student of an affiliated institution which is paying any applicable article publishing charge ("APC") for Open Access articles. Where the Submitting Author wishes to make the Work available on an Open Access basis (and intends to pay the relevant APC), the terms of reuse of such Open Access shall be governed by a Creative Commons licence – details of these licences and which Creative Commons licence will apply to this Work are set out in our licence referred to above.

Other than as permitted in any relevant BMJ Author's Self Archiving Policies, I confirm this Work has not been accepted for publication elsewhere, is not being considered for publication elsewhere and does not duplicate material already published. I confirm all authors consent to publication of this Work and authorise the granting of this licence.

For peer review only

Title: Association of Maternal Depression and Anxiety with Toddler Social-Emotional and Cognitive Development in South Africa: A prospective cohort study

Lauren C. Shuffrey*[1,2], Ayesha Sania*[1,2], Natalie H. Brito[3], Mandy Potter[4], Priscilla E. Springer[5], Maristella Lucchini[1,2], Yael K. Rayport[1,2], Carlie Du Plessis[4], Hein J. Odendaal[4], William P. Fifer[1,2,6]

[1] Department of Psychiatry, Columbia University Irving Medical Center, New York, NY 10032 USA
[2] Division of Developmental Neuroscience, New York State Psychiatric Institute, New York, NY 10032 USA
[3] Department of Applied Psychology, New York University, New York, NY, 10003 USA
[4] Department of Obstetrics and Gynaecology, Faculty of Medicine and Health Science, Stellenbosch University, Cape Town, Western Cape, South Africa 7530
[5] Department of Paediatrics and Child Health, Stellenbosch University, Cape Town, Western Cape, South Africa 7530
[6] Department of Pediatrics, Columbia University Irving Medical Center, New York, NY 10032 USA
*** denotes shared authorship**

Corresponding Author:
Lauren C. Shuffrey, PhD
Columbia University Medical Center
New York State Psychiatric Institute
1051 Riverside Drive, Unit 40
New York, New York 10032
lcg2129@cumc.columbia.edu

Financial Support: This research was supported by the Bill and Melinda Gates Foundation (WPF) and grants U01HD055154, U01HD045935, U01HD055155, and U01HD045991 issued by the *Eunice Kennedy Shriver* National Institute of Child Health and Human Development (NICHD) and grant U01AA016501 issued by the National Institute on Alcohol Abuse and Alcoholism (NIAAA). Dr. Lauren Shuffrey is supported by K99HD103910 issued by the Eunice Kennedy Shriver National Institute Of Child Health & Human Development. Dr. Ayesha Sania is supported by UH3OD023279-05S1, re-entry supplement from Office of the Director, NIH, and Office of Research on Women Health (ORWH). The content is solely the responsibility of the authors and does not necessarily represent the official views of the National Institutes of Health.

Competing Interests: The authors have no competing interests to declare.

Data availability: Data are available upon reasonable request.

Patient and Public Involvement: Patients or the public were not involved in the design, or conduct, or reporting, or dissemination plans of our research

For peer review only

Abstract:

Objective: A robust literature has identified associations between prenatal maternal depression and adverse child social-emotional and cognitive outcomes. The majority of prior research is from high-income countries despite increased reporting of perinatal depression in low and middle income countries (LMICs). Additionally, despite the comorbidity between depression and anxiety, few prior studies have examined their joint impact on child neurodevelopment. The objective of the current analysis was to examine associations between prenatal maternal depression and anxiety with child social-emotional and cognitive development in a cohort from the Western Cape Province of South Africa.

Design: Prenatal maternal depression and anxiety were measured using the Edinburgh Postnatal Depression Scale (EPDS) and the State-Trait Anxiety Scale (STAI) at 20 – 24 weeks' gestation. Child neurobehavior was assessed at age 3 using the Brief Infant Toddler Social Emotional Assessment (BITSEA) and the Bayley Scales of Infant Development Screening Test (BSID-III ST). We used linear regression models to examine the independent and joint association between prenatal maternal depression, anxiety, and child developmental outcomes.

Results: Participants consisted of 600 maternal-infant dyads (274 females; gestational age at birth: 38.89 weeks ± 2.03). Children born to mothers with both prenatal depression and trait anxiety had higher social-emotional problems (mean difference: 4.66; 95% CI 3.43, 5.90) compared to children born to mothers with no prenatal depression or trait anxiety, each condition alone, or compared to mothers with depression and state anxiety. Additionally, children born to mothers with prenatal maternal depression and trait anxiety had the greatest reduction in mean cognitive scores on the BSID-III ST (mean difference: -1.04; 95% CI -1.99, -0.08).

Conclusions: The observed association between comorbid prenatal maternal depression and chronic anxiety with subsequent child social-emotional and cognitive development underscores the need for targeting mental health support in perinatal women in LMICs to improve long-term child neurobehavioral outcomes.

For peer review only

For peer review only - http://bmjopen.bmj.com/site/about/guidelines.xhtml

Strengths and limitations of this study

- The current study included a prospective evaluation of maternal depression and anxiety symptoms during pregnancy and prospective assessment of cognitive and social-emotional outcomes within the same cohort in a large sample of South African mother-children pairs.

- Limitations include a lack of data on maternal mental health assessments postnatally and mother-child dyadic measures, which are potential mediators of the relationship between prenatal maternal depression, prenatal maternal anxiety, and child developmental outcomes.

- This study addresses a significant gap in the literature in research examining the impact of prenatal maternal psychological health on child neurobehavioral development in resource-poor communities.

Keywords: prenatal depression, prenatal anxiety; social-emotional development; cognitive development

For peer review only

Introduction

Decades of research on the early origins of behavior has promoted the concept that the prenatal environment has a profound impact on one's risk for the development of neurodevelopmental or psychiatric disorders[1 2]. Several prior studies have identified associations between prenatal maternal depression and increased risk for social-emotional problems and decrements in cognitive development. However, the majority of prior research in this domain is from high-income countries (HICs), despite increased reporting of perinatal depression in low and middle income countries (LMICs) including South Africa where an estimated 35% women report prenatal depression[3 4]. Additionally, despite the comorbidity between depression and anxiety, few prior studies have examined their joint impact on child neurodevelopment.

Several studies in HICs have identified associations between prenatal maternal depression, anxiety, and offspring behavioral, social-emotional, and cognitive development[5]. Specifically, a meta-analysis demonstrated adverse effects of prenatal maternal depression and anxiety on child social-emotional problems, with odds ratios of 1.79 and 1.50 respectively[6]. Prenatal maternal depression and anxiety are also associated with cognitive and language deficits[7 8], delayed motor development[8], emotional and behavior dysregulation[9-11], inattention and hyperactivity[12-14], and difficult temperament[15]. A more recent meta-analysis not only confirmed prior reports, but also found that the effects of perinatal maternal depression extend beyond infancy through adolescence[16].

The developmental origins of health and disease (DOHaD) model posits that maternal psychological distress during pregnancy (e.g. perceived stress, depression, anxiety, post-traumatic stress) may result in changes in hypothalamic pituitary adrenal (HPA) axis function and upregulation of inflammatory processes with downstream effects on offspring perinatal brain

For peer review only

development and behavior[17]. Prior research suggests comorbid prenatal maternal depression and anxiety may be associated with the greatest increases in maternal HPA-axis activity and differential changes in immunologic activity. Specifically, comorbid prenatal maternal depression and anxiety have been associated with a greater increase in salivary cortisol levels[18], TH1 secreted cytokines, TH2 secreted cytokines, and TH17 secreted cytokines[19] compared to either condition alone. Other research suggests cytokine profiles may differ between individuals with prenatal maternal depression and anxiety[19-21].

Risk factors may be exacerbated in LMICs such as South Africa, where both poverty and perinatal mental health disorders are highly prevalent[22 23]. Specifically, in South Africa maternal mood disorders have been linked to structural and community stressors associated with markers of poverty including less than a high school education, lack of social support, alcohol use, family stress, food insecurity, lack of partner involvement, and intimate partner violence[24-28]. There are few prior studies that have examined the impact of prenatal maternal mental health on offspring social-emotional or cognitive development in South Africa. While the few prior studies reported significant harmful effects of prenatal maternal stress or perinatal maternal depression, there is still a significant gap in the literature in research examining the impact of prenatal maternal psychological health on child neurobehavioral development in resource-poor communities[24 29 30]. Additionally, to our knowledge no prior South African studies have examined the joint effect of prenatal maternal depression and anxiety on child neurobehavioral outcomes. Research examining the long-term impact of prenatal maternal depression and anxiety on child behavioral and cognitive outcomes is critical for providing justification to local public health services for targeting mental health support in perinatal women from underserved communities.

For peer review only - http://bmjopen.bmj.com/site/about/guidelines.xhtml

The objective of the current analysis was to determine if prenatal maternal depression and state or trait anxiety were associated with child social-emotional problems or cognitive development at approximately three years of age in a South African cohort from the Western Cape.

Materials and Methods

Participants. Participants were a subset of infants with available outcome data at age 3 enrolled in the Safe Passage Study conducted by the Prenatal Alcohol and SIDS and Stillbirth (PASS) Network, a multi-center study investigating the role of prenatal exposure in risk for sudden infant death syndrome (SIDS), stillbirth, and fetal alcohol spectrum disorders. Eligibility criteria for the Safe Passage study included the ability to provide informed consent in English or Afrikaans, 16 years of age or older at the time of consent, and a gestational age between 6 weeks and 40 weeks at the time of consent based on estimated delivery date[31]. Exclusion Criteria for prenatal maternal enrollment into the Safe Passage study included planned therapeutic abortion, moving out of the catchment area prior to estimated date of delivery, and clinical judgment. Informed consent was obtained for the Safe Passage study and from a parent or legal guardian of each participant for developmental follow-up assessments. Ethical approval was obtained for both time points from the Health Research Ethics Committee of Stellenbosch University and the New York State Psychiatric Institute.

Maternal Assessments.

Maternal-infant chart abstraction, demographic, and socioeconomic measures. Maternal-infant medical charts were abstracted to obtain maternal age at delivery, gestational age at birth,

For peer review only - http://bmjopen.bmj.com/site/about/guidelines.xhtml

mode of delivery, and the infant's biological sex. Measures to collect prenatal alcohol, tobacco, and recreational drug exposure have been previously described[31 32]. Prenatal maternal alcohol and tobacco use behaviors were previously characterized using cluster analysis[33 34]. Through study specific case report forms, participants indicated demographic and socioeconomic information including race, maternal educational attainment, household crowding (persons per room in household), access to running water inside the house, prenatal care during pregnancy, and marital status.

Self-reported depression and anxiety measures. Information regarding maternal mental health during pregnancy was obtained at 20 – 24 weeks' gestation. Depressive symptoms were measured using the Edinburgh Postnatal Depression Scale (EPDS), a depression screening tool developed to specifically assess depressive symptoms in perinatal women where higher scores indicate more severe depressive symptoms[35 36]. The EPDS is widely used and has been validated in English and Afrikaans in South Africa[35 37]. Prior studies have used a cut-off score of $\geq$12 or $\geq$ 13 to be indicative of major depression within perinatal women living in South Africa[35 37]. Maternal anxiety symptoms were measured using the State-Trait Anxiety Inventory (STAI)[38], an anxiety screening tool to distinguish anxiety symptoms from depressive symptoms which has also been validated in both languages[39]. The STAI has two subscales, state anxiety which reflects the participant's current state of anxiety when completing the questionnaires and trait anxiety, which is thought to be consistent across time and reflect personality traits. In HICs, the STAI has a cut-off score of $\geq$ 40 on both the state anxiety and trait anxiety subscales to indicate a threshold for clinical levels of anxiety. Based on these prior studies, we used a cutoff of $\geq$ 13 to indicate maternal depression, a cutoff of $>$ 40 on the STAI-state subscale to indicate state anxiety, and a

For peer review only - http://bmjopen.bmj.com/site/about/guidelines.xhtml

cutoff of > 40 on the STAI-trait subscale to indicate trait anxiety.

Toddler Developmental Assessments.

Bayley Scales of Infant Development III Screening Test. The Bayley Scales of Infant Development III Screening Test (BSID-III ST) were designed as a rapid assessment of cognitive, language, and motor functioning in infants and young children in order to determine if a child's development is within normal limits and identify risk for developmental delay. The BSID-III ST has high test-retest reliability: Cognitive (0.85), Receptive Language (0.88), Expressive Language (0.88), Fine Motor (0.82), and Gross Motor (0.86). Although the BSID-III ST does not identify degree of impairment, the cut-off points indicate whether a child shows competence in age-appropriate tasks, evidence of emerging age-appropriate skills, and evidence of being at risk for developmental delay. The BSID has been validated and widely used throughout South Africa[40 41].

Brief Infant Toddler Social Emotional Assessment (BITSEA). The Brief Infant-Toddler Social and Emotional Assessment (BITSEA) is a 42-item parental report measure of social-emotional development, behavioral problems, and delays in competence[42]. Domains assessed within the BITSEA include: externalizing (activity/impulsivity, aggression/defiance, peer aggression), internalizing (depression/withdrawal, anxiety, separation distress, inhibition to novelty), dysregulation (sleep, negative emotionality, eating, sensory sensitivity), and competence (compliance, attention, imitation/play, mastery motivation, empathy, and pro-social peer relations)[42]. Findings from the BITSEA validation study provide preliminary support for the BITSEA as a reliable and valid brief screener for infant-toddler social-emotional and behavioral problems in addition to delays in competence[43]. When used in socioeconomically and ethnically

For peer review only - http://bmjopen.bmj.com/site/about/guidelines.xhtml

diverse community-based populations, the BITSEA demonstrated excellent test-retest reliability and good inter-rater agreement between parents[42].

Statistical Analyses. Using multiple linear regression models, we estimated independent and joint effects of maternal depression and state and trait anxiety on social-emotional problem, social emotional competence, and cognitive development scores. Two, separate four-level categorical prenatal maternal mental health variables were created to assess the impact of prenatal maternal depression, trait anxiety, and state anxiety. We created a prenatal maternal depression and trait anxiety variable with four categories: (1) *No Prenatal Depression or Trait Anxiety* (n=199; 33.17%, Reference Category), (2) *Prenatal Depression Only* (106; 17.67%), (3) *Prenatal Trait Anxiety Only* (n=68; 11.33%) and (4) *Prenatal Maternal Depression and Trait Anxiety* (n=227; 37.83%) (Table 1). In separate models we additionally examined the independent and joint effects of prenatal maternal depression and state anxiety. We created a prenatal maternal depression and state anxiety variable with four categories: (1) *No Prenatal Depression or State Anxiety* (n=248; 41.33%; Reference Category), (2) *Prenatal Depression Only* (n=237; 39.50%), (3) *Prenatal State Anxiety Only* (n=19; 3.17%) and (4) *Prenatal Maternal Depression and State Anxiety* (n=96; 16%) (Table 1). For each regression model, either *No Prenatal Maternal Depression or Trait Anxiety* or *No Prenatal Maternal Depression and State Anxiety* was set as the reference category. Minimally adjusted models included sex, gestational age at birth, and age at follow up as covariates. Fully adjusted models additionally controlled for prenatal maternal alcohol use, prenatal maternal tobacco use, maternal employment status at delivery, maternal educational attainment at delivery, parity, and the household crowding index. We used missing indicator methods and median imputation to

For peer review only - http://bmjopen.bmj.com/site/about/guidelines.xhtml

account for missing categorial and continuous covariate data, respectively (described in Table 1). All analyses were performed in SAS software version 9.4 (SAS Institute, Cary NC).

Results

Maternal and Child Demographic Characteristics. The participants included in the present analysis consisted of mothers and their infant born between April 2014 and August 2015 from the Western Cape Province of South Africa who participated in a follow-up study to examine social-emotional development and cognitive development at approximately three years of age. A total of n=18 mother-infant dyads were excluded due to missing maternal prenatal mental health data. The final sample consisted of 600 maternal-infant dyads (274 females; gestational age at birth: 38.89 weeks ± 2.03) (Table 1).

Child Social-Emotional Development. Based on the BITSEA problem scale percentile rank score of 26 or higher, 51% of children (306/600) were classified as having a "possible problem". Based on the BITSEA competence scale percentile rank score of 15 of lower, 5% (30/600) of children were classified in the "possible deficit/delay range" for social competencies (Table 2). There were no significant sex differences in social-emotional problems on the BITSEA, however girls had significantly higher social-emotional competence compared to boys (mean difference: 0.38, CI: 0.05, 0.71, p = 0.03)

Association between Prenatal Maternal Depression, Trait Anxiety, and Child Social-Emotional Development. Compared to children born to mothers with no prenatal depression or trait anxiety, children born to mothers with prenatal depression and trait anxiety had higher social-emotional problems (mean difference: 4.66; 95% CI 3.43, 5.90), followed by prenatal maternal trait anxiety only (mean difference: 3.87; 95% CI 2.07, 5.66), and finally prenatal maternal depression only (mean difference: 2.76; 95% CI 1.23, 4.29) in minimally adjusted

For peer review only - http://bmjopen.bmj.com/site/about/guidelines.xhtml

models. These associations remained significant in fully adjusted models where similarly comorbid prenatal maternal depression and trait anxiety group was associated with the highest child social-emotional problems (mean difference: 4.33; 95% CI 2.90, 5.67), followed by prenatal maternal trait anxiety only (mean difference: 3.23; 95% CI 1.19, 5.27), with the smallest mean difference for prenatal maternal depression only group (mean difference: 2.64; 95% CI 1.02, 4.27) as compared to the no prenatal depression or trait anxiety group (Figure 1). Additional significant predictors in the multivariate models were parity of 3 or greater which was associated with lower social-emotional problems (mean difference: -3.02, 95% CI -4.63, -1.40) and low continuous smoking during pregnancy which was associated with higher social-emotional problems (mean difference: 1.39, 95% CI 0.039, 2.74). There were no significant associations between prenatal maternal depression, trait anxiety, and child social-emotional competence.

Association between Prenatal Maternal Depression, State Anxiety, and Child Social-Emotional Development. Compared to children born to mothers with no prenatal depression or state anxiety, children born to mothers with comorbid prenatal depression and state anxiety had higher social-emotional problems (mean difference: 4.29; 95% CI 2.73, 5.84). Children born to mothers with prenatal depression only also had higher social-emotional problems compared to mothers with no prenatal depression or state anxiety (mean difference: 2.71: 95% CI 1.51, 3.88). These associations remained significant in fully adjusted models (prenatal depression and state anxiety: 3.90 mean increase (95% CI 2.19, 5.60); prenatal depression only: 2.58 mean increase (95% CI 1.34, 3.82)) (Figure 1). Additional significant predictors in the multivariate models were parity of 3 or greater which was associated with lower social-emotional problems (mean difference: -3.22, 95% CI -4.86, -1.58), low continuous smoking during pregnancy which was

For peer review only - http://bmjopen.bmj.com/site/about/guidelines.xhtml
For peer review only

associated with higher social-emotional problems (mean difference: 1.44; 95% CI 0.075, 2.81), and finally less than a high school education (some primary school only) which was associated with higher social-emotional problems (mean difference: 3.47; 95% CI 0.248, 6.70). However, there was no significant association between prenatal state anxiety only and child social-emotional problems on the BITSEA. There were also no significant associations between prenatal maternal depression, state anxiety, and social-emotional competence.

Child Cognitive, Language, and Motor Development. Based on normative cognitive cutoff scores defined by the BSID-III ST, 4% of children (24/600) were classified as at-risk (Table 2). Risk classification percentages were similar across all subdomains, expressive language: 4% , receptive language: 6%, gross motor: 4%, and fine motor: 3%. There were no significant sex differences in child cognitive scores.

Association between Prenatal Maternal Depression, Trait Anxiety, and Child Cognitive, Language, and Motor Development. Compared to children born to mothers with no prenatal depression or trait anxiety, children born to mothers with comorbid prenatal depression and trait anxiety had lower cognitive scores on the BSID-III ST (mean difference: -1.04; 95% CI -1.99, -0.08). Results remained significant in the fully adjusted model (mean difference: -1.11; 95% CI -2.13, -0.09) (Figure 2) and in posthoc analyses where we additionally controlled for language of administration for the BSID-III ST (mean difference: -0.51, 95% CI -0.99, -0.042). Children who were assessed on the BSID-III ST in Afrikaans (mean difference: -1.00, 95% CI -1.48, -0.52) or who were assessed in mixed English and Afrikaans (mean difference: -1.30, 95% CI -2.07, -0.54) has significantly lower cognitive scores compared to children assessed in English. Low continuous prenatal maternal alcohol use was also associated with lower cognitive scores (mean difference: -1.30; 95% CI -2.36, -0.24).

For peer review only - http://bmjopen.bmj.com/site/about/guidelines.xhtml

Association between Prenatal Maternal Depression, State Anxiety, and Child Cognitive, Language, and Motor Development. Compared to children born to mothers with neither prenatal depression nor trait anxiety, there was no significant association between cognitive scores for children born to mothers with prenatal depression only, prenatal trait anxiety only, or combined prenatal depression and trait anxiety in either the minimally or fully adjusted models.

Discussion

In summary, we found the greatest increase in child social-emotional problems in children born to women with comorbid prenatal depression and trait anxiety compared to women with no prenatal depression or trait anxiety, prenatal depression alone, or prenatal trait anxiety alone. We additionally found a significant association between comorbid prenatal maternal depression and state anxiety on higher child social-emotional problems; however, we found no association between prenatal maternal state anxiety in the absence of prenatal maternal depression on child social-emotional problems. Finally, we reported children born to mothers with prenatal maternal depression and trait anxiety had lower cognitive scores on the BSID-III ST, but we did not find an association between prenatal maternal depression alone on cognitive outcomes at 3-years of age.

Our finding linking prenatal maternal depression and anxiety to social-emotional risk parallels two recent South African studies linking increased prenatal maternal stressors to higher behavioral problems in children with an odds ratio of 2.52, but not before 4 years of age[29] and increased perinatal depression to aggressive behaviors at 60 months of age[24]. Our findings are also largely consistent with a recent meta-analysis of studies predominately conducted in HICs, which found prenatal maternal depression and anxiety were associated with higher social-

For peer review only - http://bmjopen.bmj.com/site/about/guidelines.xhtml

emotional problems in offspring with larger effect sizes for prenatal maternal depression (OR 1.79; 95% CI 1.61 - 1.99) compared to prenatal maternal anxiety (OR 1.50, 95% CI, 1.36 - 1.64)[6]. We found the greatest increase in child social-emotional problems in children born to women with comorbid prenatal depression and trait anxiety and no effect of prenatal maternal state anxiety in the absence of prenatal maternal depression on child social-emotional problems. Our results are suggestive that chronic anxiety measured via trait anxiety may be more predictive of child social-emotional outcomes than a single measurement of concurrent anxiety during pregnancy.

A prior meta-analysis[6] also found stronger effects of prenatal maternal depression and anxiety on child social-emotional outcomes when sociodemographic risk factors such as low-income, lower levels of parental education, or single family households, were highest[6]. Similarly, we found that lower levels of maternal education, low continuous tobacco use during pregnancy, and low continuous alcohol use during pregnancy were associated with higher social-emotional problems in children at three years of age. Intriguingly, maternal parity of 3 or greater was protective and associated with lower social-emotional problems which may be due to reduced depression and anxiety levels in women with higher parity, changes in the perception of their child's behavior due to having multiple children, or additional social opportunities during sibling interactions[44 45].

The Drakenstein Child Health Study based in the Western Cape found approximately 50% of the overall sample (369/731) were categorized as having cognitive delay at two years of age based on cutoffs defined by United States (US) normative data[30]. Better cognitive outcomes were associated with higher maternal education, older child age, a primigravid mother, and higher socioeconomic status whereas prenatal maternal depression was associated with a 1.03

For peer review only - http://bmjopen.bmj.com/site/about/guidelines.xhtml

SD (95% CI -1.94 to -0.12) reduction in cognitive scores on the Bayley Scales of Infant and Toddler Development at two years of age[30]. Similar to the Drakenstein Child Health Study[30], we found children born to mothers with prenatal maternal depression and trait anxiety had lower cognitive scores on the BSID-III ST compared to the no prenatal maternal depression or trait anxiety group. In contrast, we did not find an association between prenatal maternal depression alone on cognitive outcomes at 3-years of age. However, since we did not utilize the full BSID-III and only administered the BSID-III ST, it is difficult to directly compare our results to The Drakenstein Child Health study findings. Moreover, both studies relied on US normative data to define cutoff scores.

More recently, a large home-visiting intervention study based in Cape Town examined the effect of prenatal maternal depression only, postnatal maternal depression only (birth – 60 months), or recurrent pre- and postnatal maternal depression. This study also accounted for several other risk factors including intimate partner violence, HIV status, and alcohol use on child social behaviors, language skills, and cognitive development. No associations were found between maternal depression at any time point with children's language or cognitive development at 36 or 60 months of age[24]. However, children of never depressed mothers had lower aggressive behaviors on the Child Behavior Checklist at 60 months of age than children of mothers with postnatal depression only or perinatal depression[24].

There are several biological mechanisms which can explain prior studies and our current findings linking prenatal maternal depression and anxiety with child social-emotional behaviors and cognitive development such as increased prenatal maternal inflammation, increased cortisol production, and/or epigenetic changes[46-55]. Pregnancy is associated with changes in inflammatory processes and increased placental cortisol production with reduced maternal HPA axis sensitivity

For peer review only - http://bmjopen.bmj.com/site/about/guidelines.xhtml

to stress[46 47]. However, prenatal maternal depression may upregulate inflammatory processes and/or cortisol production. Additionally, prior research found women with comorbid depression and anxiety have the greatest increases in salivary cortisol levels[18]. Maternal cortisol can cross the placenta resulting in increased inflammation[46 47] and/or affect the developmental of limbic regions, which are associated with social and emotional processes[56]. In animal models, increased proinflammatory proteins have been associated with widespread changes in perinatal brain development such as with volume reductions in gray and white matter, decreased density of GABAergic neurons, reduced synaptic pruning, and network dysfunction with potential downstream effects on neurobehavioral development[48-55]. Other studies examining the intergenerational transmission of trauma have demonstrated transgenerational epigenetic changes in animal models[57]. Taken together, prior research suggests multiple overlapping pathways by which prenatal maternal mood can affect offspring brain-behavioral development.

While we report an association of prenatal maternal depression and anxiety with child social-emotional and cognitive development from a prospective cohort study with a fairly large sample of participants, there are several methodological and contextual limitations within the current study that are worth noting. First, it is important to note that the reliance on maternal-report measures to characterize child social-emotional development is a limitation in the majority of research to date, including the present study. The reliance on maternal reporting of child social-emotional development may be influenced by factors such as maternal mood or education. An additional limitation was the use of the BSID-III screening test in the current study to measure cognitive development, which is based on US normative data. Future studies should consider objective measures of child social-emotional development through observational or

For peer review only - http://bmjopen.bmj.com/site/about/guidelines.xhtml

behavioral coding paradigms in addition to utilizing objective cognitive developmental assessments of with normative data in South African children.

There are also several unmeasured contextual factors which could affect our findings. For example, although prenatal maternal and child nutrition are known to affect child neurobehavioral development, we lacked measures of prenatal maternal nutrition, prenatal maternal micronutrient deficiencies, prenatal and postnatal household food insecurity, and information regarding child nutrition. Additionally, prior studies have emphasized the importance of measuring childhood trauma, maternal stress, and social support within communities with several heterogeneous risk factors[3], these measures were not collected as part of the original NIH Safe Passage Study or our recent follow-up study. Therefore, we could not examine their effects or consider maternal social support as a potential moderator of resilient neurodevelopmental outcomes. There is also robust literature examining both postpartum depression (PPD) and the early mother-infant relation in shaping child outcomes. Two prior studies in South Africa demonstrated maternal intrusiveness and coerciveness mediated the association between maternal PPD and early childhood attachment[58 59]. In the current study, we did not collect data postnatally between birth and three years of age. Additionally, we did not collect postnatal information on maternal depression, anxiety, or stress. Therefore, due to this methodological limitation we cannot draw conclusions regarding the combined effect of the pre- and postnatal environment on child neurodevelopmental outcomes, nor can we assess potential interaction effects between pre- and postnatal maternal mood on child social-emotional and cognitive outcomes. Finally, our results may not generalize to all South African populations where HIV rates can be as high as 35% since our cohort only included one woman with HIV. Strengths of the current analyses include our large sample size, evaluation of both cognitive and

For peer review only - http://bmjopen.bmj.com/site/about/guidelines.xhtml

For peer review only

social-emotional outcomes within the same cohort, finally the detailed prospective collection of both depression and anxiety symptoms during pregnancy[31] in addition to other potential confounders.

Our results suggest comorbid prenatal maternal depression and chronic anxiety have a greater impact on child social-emotional and cognitive development than either condition alone or than comorbid prenatal maternal depression and transitory anxiety during pregnancy. These findings are supported by a robust literature within the DOHaD framework linking perturbations in the gestational environment to later neurodevelopmental or psychiatric sequelae. Our results also lend support for future intervention studies aimed at perinatal mental health interventions targeting maternal depressive and anxiety symptoms to improve long-term child social-emotional and cognitive developmental outcomes in low-resource communities.

For peer review only

For peer review only - http://bmjopen.bmj.com/site/about/guidelines.xhtml

Table 1 - Sociodemographic characteristics		
	Mean ± SD or N (%)	N and Percent Missing (%)
**Maternal Characteristics**		
Maternal Age (years)	25.26 ± 5.91	0 (0%)
Maternal Body Mass Index (BMI)		0 (0%)
BMI <18.5 (kg/m2)	62 (10.33%)	
BMI 18.5-25 (kg/m2)	282 (47%)	
BMI 25-30 (kg/m2)	132 (22%)	
BMI >30 (kg/m2)	124 (20.67%)	
Parity		0 (0%)
Parity <1	218 (36.33%)	
Parity =1	196 (32.67%)	
Parity =2	103 (17.17%)	
Parity >=3	83 (13.83%)	
Antenatal care visits		0 (0%)
antenatal care visit <3	49 (8.17%)	
antenatal care visit 3-6	389 (64.83%)	
antenatal care visit >6	162 (27%)	
Cesarean Section	106 (17.70%)	1 (0.1%)
Education		0 (0%)
Some primary school	43 (7.167%)	
Some high school	405 (67.57%)	
Completed high school	119 (19.83%)	
Beyond high school	33 (5.5%)	
Married	290 (48.33%)	0 (0%)
Employed	172 (28.67%)	0 (0%)
Adjusted Household Crowding	1.56 ± 0.72	2 (0.33%)
Depression (Edinburgh >=13)	333 (55%)	0 (0%)
Anxiety (State-Trait Anxiety Inventory >=40)	115 (19.17%)	0 (0%)
Maternal Prenatal Alcohol Use Cluster Groups		0 (0%)
Non drinking group: 0 standard drinks/trimester	245 (46.93%)	
Moderate-high continuous drinking group:	122 (23.37%)	
Standard drinks in Trimester 1	27 ± 39	
Standard drinks in Trimester 2	17 ± 25	
Standard drinks in Trimester 3	9.4 ± 15	
Binge drinking events ($\geq$4 drinks/day) Trimester 1	2.7 ± 4	

For peer review only - http://bmjopen.bmj.com/site/about/guidelines.xhtml

Binge drinking events (≥4 drinks/day) Trimester 2	1.7 ± 2.8	
Binge drinking events (≥4 drinks/day) Trimester 3	0.89 ± 1.7	
Low continuous drinking	26 (4.98%)	
Standard drinks in Trimester 1	1.4 ± 2.5	
Standard drinks in Trimester 2	4 ± 2.8	
Standard drinks in Trimester 3	0.62 ± 1.1	
Binge drinking events (≥4 drinks/day) Trimester 1	0.067 ± 0.25	
Binge drinking events (≥4 drinks/day) Trimester 2	0.30 ± 0.46	
Binge drinking events (≥4 drinks/day) Trimester 3	0 ± 0	
Quit early drinking	129 (24.71%)	
Standard drinks in Trimester 1	8.5 ± 6.5	
Standard drinks in Trimester 2	0.31 ± 0.87	
Standard drinks in Trimester 3	0.056 ± 0.31	
Binge drinking events (≥4 drinks/day) Trimester 1	0.84 ± 0.82	
Binge drinking events (≥4 drinks/day) Trimester 2	0 ± 0	
Binge drinking events (≥4 drinks/day) Trimester 3	0 ± 0	
Maternal Prenatal Tobacco Use		2 (0.33%)
Non smoking (0 cigarettes/trimester)	227 (37.97%)	
Moderate-high continuous smoking	132 (22.07%)	
Average Cigarettes in Trimester 1	45 ± 20	
Average Cigarettes in Trimester 2	50 ± 27	
Average Cigarettes in Trimester 3	48 ± 25	
Low continuous smoking	222 (37.12%)	
Average Cigarettes in Trimester 1	16 ± 9.4	
Average Cigarettes in Trimester 2	16 ± 9.7	
Average Cigarettes in Trimester 3	16 ± 10	
Quit early smoking	17 (2.84%)	
Average Cigarettes in Trimester 1	11 ± 7.5	
Average Cigarettes in Trimester 2	0.15 ± 0.32	
Average Cigarettes in Trimester 3	0.079 ± 0.11	
Raw Maternal Edinburgh Score	12.99 ± 5.73	0 (0%)
Raw Maternal State Anxiety Score	31.23 ± 10.24	0 (0%)
Raw Maternal Trait Anxiety Score	40.63 ± 10.63	0 (0%)
Depression - Trait Anxiety Groups		0 (0%)
No depression or trait anxiety	199 (33.17%)	
Depression alone	106 (17.67%)	
Trait anxiety alone	68 (11.33%)	
Depression and trait anxiety	227 (37.83%)	
Depression - State Anxiety Groups		0 (0%)
No depression or state anxiety	248 (41.33%)	

For peer review only

	Depression alone	237 (39.50%)	
	State anxiety alone	19 (3.17%)	
	Depression and state anxiety	96 (16%)	
HIV status			
	Tested for HIV	600 (100%)	
	HIV positive	1 (0.1%)	
**Infant Characteristics**			
Infant Sex			0 (0%)
	Male	326 (54.33%)	
	Female	274 (45.67%)	
Gestational age at birth (weeks)		38.89 ± 2.03	0 (0%)
Infant Birth weight (grams)		2980.65 ± 564.65	2 (0.33%)
Follow-up age (months)		38.29 ± 2.96	0 (0%)
Adjusted follow-up age (months)		38.14 ± 0.016	0 (0%)

Table 2. Neurodevelopmental Outcome Raw Scores and At-risk Groups

BSID-III Screening Test Language of Administration		
	English Only	177 (30%)
	Afrikaans Only	360 (62%)
	Mixed English and Afrikaans	48 (8%)
BSID-III Screening Test Scores		
	Gross motor	25.47 ± 4.54
	Fine motor	23.55 ± 4.08
	Cognitive	27.77 ± 4.99
	Problem solving	13.40 ± 6.77
	Receptive language	21.69 ± 5.10
	Expressive language	21.27 ± 3.78
At-Risk Categories		
	Gross motor	21 (4%)
	Fine motor	18 (3%)
	Cognitive	24 (4%)
	Receptive language	33 (6%)
	Expressive language	25 (4%)
Brief Infant Toddler Social-Emotional Assessment (BITSEA)		
	Social Emotional Problem	13.40 ± 6.77
	Competence	19.56 ± 2.07
At-Risk Categories		
	Social Emotional Problems	306 (51%)

For peer review only

For peer review only - http://bmjopen.bmj.com/site/about/guidelines.xhtml

Social-Emotional Competence	30 (5%)

Figure Legends

Figure 1. Association between prenatal maternal depression, prenatal maternal anxiety, and child social-emotional problems measured by the BITSEA. Each line plot depicts the mean difference and their confidence interval for child social-emotional problems (x-axis) for each prenatal maternal mental health group (y-axis). Either no prenatal maternal depression and state anxiety or no prenatal maternal depression and trait anxiety were the reference groups. Models were adjusted for sex, gestational age at birth, age at follow-up, prenatal maternal alcohol use, prenatal maternal tobacco use, maternal employment status at delivery, maternal educational attainment at delivery, parity, and the household crowding index.

Figure 2. Association between prenatal maternal depression, prenatal maternal anxiety, and child cognitive development measured by the BSID-III Screening Test. Each line plot depicts the mean difference and their confidence interval for child cognitive development (x-axis) for each prenatal maternal mental health group (y-axis). Either no prenatal maternal depression and state anxiety or no prenatal maternal depression and trait anxiety were the reference groups. Models were adjusted for sex, gestational age at birth, age at follow-up, prenatal maternal alcohol use, prenatal maternal tobacco use, maternal employment status at delivery, maternal educational attainment at delivery, parity, and the household crowding index.

References

1. O'Donnell KJ, Meaney MJ. Fetal Origins of Mental Health: The Developmental Origins of Health and Disease Hypothesis. *Am J Psychiatry* 2017;174(4):319-28. doi: 10.1176/appi.ajp.2016.16020138 [published Online First: 2016/11/15]
2. Hofer MA. The Roots of Human Behavior: An Introduction to the Psychobiology of Early Development. San Francisco, CA: W. H. Freeman & Company 1981.
3. Stein DJ, Koen N, Donald KA, et al. Investigating the psychosocial determinants of child health in Africa: The Drakenstein Child Health Study. *J Neurosci Methods* 2015;252:27-35. doi: 10.1016/j.jneumeth.2015.03.016 [published Online First: 2015/03/24]
4. Herba CM, Glover V, Ramchandani PG, et al. Maternal depression and mental health in early childhood: an examination of underlying mechanisms in low-income and middle-income countries. *Lancet Psychiatry* 2016;3(10):983-92. doi: 10.1016/S2215-0366(16)30148-1 [published Online First: 2016/09/22]
5. Monk C, Lugo-Candelas C, Trumpff C. Prenatal Developmental Origins of Future Psychopathology: Mechanisms and Pathways. *Annu Rev Clin Psychol* 2019;15:317-44. doi: 10.1146/annurev-clinpsy-050718-095539 [published Online First: 2019/02/24]

For peer review only - http://bmjopen.bmj.com/site/about/guidelines.xhtml

6. Madigan S, Oatley H, Racine N, et al. A Meta-Analysis of Maternal Prenatal Depression and Anxiety on Child Socioemotional Development. *J Am Acad Child Adolesc Psychiatry* 2018;57(9):645-57 e8. doi: 10.1016/j.jaac.2018.06.012
7. King S, Laplante DP. The effects of prenatal maternal stress on children's cognitive development: Project Ice Storm. *Stress* 2005;8(1):35-45. doi: 10.1080/10253890500108391
8. Huizink AC, Robles de Medina PG, Mulder EJ, et al. Stress during pregnancy is associated with developmental outcome in infancy. *J Child Psychol Psychiatry* 2003;44(6):810-8. doi: 10.1111/1469-7610.00166 [published Online First: 2003/09/10]
9. Leis JA, Heron J, Stuart EA, et al. Associations between maternal mental health and child emotional and behavioral problems: does prenatal mental health matter? *J Abnorm Child Psychol* 2014;42(1):161-71. doi: 10.1007/s10802-013-9766-4
10. O'Connor TG, Heron J, Golding J, et al. Maternal antenatal anxiety and children's behavioural/emotional problems at 4 years. Report from the Avon Longitudinal Study of Parents and Children. *Br J Psychiatry* 2002;180:502-8.
11. O'Donnell KJ, Glover V, Barker ED, et al. The persisting effect of maternal mood in pregnancy on childhood psychopathology. *Dev Psychopathol* 2014;26(2):393-403. doi: 10.1017/S0954579414000029
12. Loomans EM, van der Stelt O, van Eijsden M, et al. Antenatal maternal anxiety is associated with problem behaviour at age five. *Early Hum Dev* 2011;87(8):565-70. doi: 10.1016/j.earlhumdev.2011.04.014
13. Rice F, Harold GT, Boivin J, et al. The links between prenatal stress and offspring development and psychopathology: disentangling environmental and inherited influences. *Psychol Med* 2010;40(2):335-45. doi: 10.1017/S0033291709005911
14. Ronald A, Pennell CE, Whitehouse AJ. Prenatal Maternal Stress Associated with ADHD and Autistic Traits in early Childhood. *Front Psychol* 2010;1:223. doi: 10.3389/fpsyg.2010.00223 [published Online First: 2010/01/01]
15. Lereya ST, Wolke D. Prenatal family adversity and maternal mental health and vulnerability to peer victimisation at school. *J Child Psychol Psychiatry* 2013;54(6):644-52. doi: 10.1111/jcpp.12012
16. Rogers A, Obst S, Teague SJ, et al. Association Between Maternal Perinatal Depression and Anxiety and Child and Adolescent Development: A Meta-analysis. *JAMA Pediatr* 2020;174(11):1082-92. doi: 10.1001/jamapediatrics.2020.2910 [published Online First: 2020/09/15]
17. Van den Bergh BRH, van den Heuvel MI, Lahti M, et al. Prenatal developmental origins of behavior and mental health: The influence of maternal stress in pregnancy. *Neurosci Biobehav Rev* 2017 doi: 10.1016/j.neubiorev.2017.07.003 [published Online First: 2017/08/02]
18. Evans LM, Myers MM, Monk C. Pregnant women's cortisol is elevated with anxiety and depression - but only when comorbid. *Arch Womens Ment Health* 2008;11(3):239-48. doi: 10.1007/s00737-008-0019-4 [published Online First: 2008/05/22]
19. Leff Gelman P, Mancilla-Herrera I, Flores-Ramos M, et al. The cytokine profile of women with severe anxiety and depression during pregnancy. *BMC Psychiatry* 2019;19(1):104. doi: 10.1186/s12888-019-2087-6 [published Online First: 2019/04/05]
20. Osborne LM, Yenokyan G, Fei K, et al. Innate immune activation and depressive and anxious symptoms across the peripartum: An exploratory study.

For peer review only - http://bmjopen.bmj.com/site/about/guidelines.xhtml

Psychoneuroendocrinology 2019;99:80-86. doi: 10.1016/j.psyneuen.2018.08.038 [published Online First: 2018/09/09]

21. Osborne LM, Brar A, Klein SL. The role of Th17 cells in the pathophysiology of pregnancy and perinatal mood and anxiety disorders. *Brain Behav Immun* 2019;76:7-16. doi: 10.1016/j.bbi.2018.11.015 [published Online First: 2018/11/23]
22. Eshetu EB, Woldesenbet SA. Are there particular social determinants of health for the world's poorest countries? *African Health Sciences* 2011;11(1):108 - 15.
23. Davies T, Rahman A, Lund C. Psychotherapy for perinatal mental disorders in low- and middle-income countries. In Global Mental Health in Practice, Global Mental Health and Psychotherapy: Academic Press, 2019:301 - 19.
24. Gordon S, Rotheram-Fuller E, Rezvan P, et al. Maternal depressed mood and child development over the first five years of life in South Africa. *J Affect Disord* 2021;294:346-56. doi: 10.1016/j.jad.2021.07.027 [published Online First: 2021/07/28]
25. Heyningen TV, Myer L, Onah M, et al. Antenatal depression and adversity in urban South Africa. *J Affect Disord* 2016;203:121-29. doi: 10.1016/j.jad.2016.05.052 [published Online First: 2016/06/11]
26. Redinger S, Norris SA, Pearson RM, et al. First trimester antenatal depression and anxiety: prevalence and associated factors in an urban population in Soweto, South Africa. *J Dev Orig Health Dis* 2018;9(1):30-40. doi: 10.1017/S204017441700071X [published Online First: 2017/09/08]
27. Redinger S, Pearson RM, Houle B, et al. Antenatal depression and anxiety across pregnancy in urban South Africa. *J Affect Disord* 2020;277:296-305. doi: 10.1016/j.jad.2020.08.010 [published Online First: 2020/08/29]
28. Drysdale RE, Slemming W, Makusha T, et al. Father involvement, maternal depression and child nutritional outcomes in Soweto, South Africa. *Matern Child Nutr* 2021;17 Suppl 1:e13177. doi: 10.1111/mcn.13177 [published Online First: 2021/07/10]
29. Ramchandani PG, Richter LM, Norris SA, et al. Maternal prenatal stress and later child behavioral problems in an urban South African setting. *J Am Acad Child Adolesc Psychiatry* 2010;49(3):239-47. [published Online First: 2010/04/23]
30. Donald KA, Wedderburn CJ, Barnett W, et al. Risk and protective factors for child development: An observational South African birth cohort. *PLoS Med* 2019;16(9):e1002920. doi: 10.1371/journal.pmed.1002920 [published Online First: 2019/09/29]
31. Dukes KA, Burd L, Elliott AJ, et al. The Safe Passage Study: Design, Methods, Recruitment, and Follow-Up Approach. *Paediatr Perinat Ep* 2014;28(5):455-65. doi: 10.1111/ppe.12136
32. Dukes K, Tripp T, Petersen J, et al. A modified Timeline Followback assessment to capture alcohol exposure in pregnant women: Application in the Safe Passage Study. *Alcohol* 2017;62:17-27. doi: 10.1016/j.alcohol.2017.02.174
33. Pini N, Shuffrey, LC., Lucchini, M., Sania, A., Nelson, ME., Odendaal, HJ., Fifer, WP., Myers, MM., Elliott, AJ. Characterization of Alcohol Consumption during Pregnancy in the Safe Passage Study. Conf Proc IEEE Eng Med Biol Soc. Berlin, Germany.
34. Shuffrey LC, Myers MM, Isler JR, et al. Association Between Prenatal Exposure to Alcohol and Tobacco and Neonatal Brain Activity: Results From the Safe Passage Study. *JAMA Netw Open* 2020;3(5):e204714. doi: 10.1001/jamanetworkopen.2020.4714 [published Online First: 2020/05/13]

For peer review only - http://bmjopen.bmj.com/site/about/guidelines.xhtml

35. Lawrie TA, Hofmeyr GJ, de Jager M, et al. Validation of the Edinburgh Postnatal Depression Scale on a cohort of South African women. *S Afr Med J* 1998;88(10):1340-4. [published Online First: 1998/11/10]
36. Alvarado R, Jadresic E, Guajardo V, et al. First validation of a Spanish-translated version of the Edinburgh postnatal depression scale (EPDS) for use in pregnant women. A Chilean study. *Arch Womens Ment Health* 2015;18(4):607-12. doi: 10.1007/s00737-014-0466-z [published Online First: 2014/10/11]
37. Hartley M, Tomlinson M, Greco E, et al. Depressed mood in pregnancy: prevalence and correlates in two Cape Town peri-urban settlements. *Reprod Health* 2011;8:9. doi: 10.1186/1742-4755-8-9 [published Online First: 2011/05/04]
38. Spielberger CD. Manual for the State-Trait Anxiety Inventory: STAI. Palo Alto, CA: Consulting Psychologists Press, 1983.
39. Pretorius TB, Norman AM. Psychometric Data on the Statistics Anxiety Scale for a Sample of South-African Students. *Educational and Psychological Measurement* 1992;52(4):933-37. doi: Doi 10.1177/0013164492052004015
40. Rademeyer V, Jacklin L. A study to evaluate the performance of black South African urban infants on the Bayley Scales of Infant Development III. *South African Journal of Child Health* 2013;7(2):54-59.
41. Ballot DE, Ramdin T, Rakotsoane D, et al. Use of the Bayley Scales of Infant and Toddler Development, Third Edition, to Assess Developmental Outcome in Infants and Young Children in an Urban Setting in South Africa. *Int Sch Res Notices* 2017;2017:1631760. doi: 10.1155/2017/1631760 [published Online First: 2017/08/25]
42. Gowan B, Carter MJ, Carter AS. ITSEA/BITSEA: Infant-Toddler and Brief Infant-Toddler Social and Emotional Assessment. *The Psychological Corporation* 2006
43. Kruizinga I, Visser JC, van Batenburg-Eddes T, et al. Screening for autism spectrum disorders with the brief infant-toddler social and emotional assessment. *PLoS One* 2014;9(5):e97630. doi: 10.1371/journal.pone.0097630 [published Online First: 2014/05/24]
44. Sang S, Nelson, JA. The effect of siblings on children's social skills and perspective taking. *Inf Child Dev* 2017:26:e2023.
45. Downey DB CD, Yucel D. Number of Siblings and Social Skills Revisited Among American Fifth Graders. *Journal of Family Issues* 2015:36(2):273-96. doi: 10.1177/0192513X13507569
46. Ghassabian A, Albert PS, Hornig M, et al. Gestational cytokine concentrations and neurocognitive development at 7 years. *Transl Psychiat* 2018;8 doi: ARTN 64
10.1038/s41398-018-0112-z
47. Ruiz RJ, Avant KC. Effects of maternal prenatal stress on infant outcomes - A synthesis of the literature. *Adv Nurs Sci* 2005;28(4):345-55. doi: Doi 10.1097/00012272-200510000-00006
48. Short SJ, Lubach GR, Karasin AI, et al. Maternal influenza infection during pregnancy impacts postnatal brain development in the rhesus monkey. *Biol Psychiatry* 2010;67(10):965-73. doi: 10.1016/j.biopsych.2009.11.026 [published Online First: 2010/01/19]
49. Paolicelli RC, Bolasco G, Pagani F, et al. Synaptic pruning by microglia is necessary for normal brain development. *Science* 2011;333(6048):1456-8. doi: 10.1126/science.1202529 [published Online First: 2011/07/23]

For peer review only

50. Bergdolt L, Dunaevsky A. Brain changes in a maternal immune activation model of neurodevelopmental brain disorders. *Prog Neurobiol* 2019;175:1-19. doi: 10.1016/j.pneurobio.2018.12.002 [published Online First: 2018/12/28]
51. Meyer U. Neurodevelopmental Resilience and Susceptibility to Maternal Immune Activation. *Trends Neurosci* 2019;42(11):793-806. doi: 10.1016/j.tins.2019.08.001 [published Online First: 2019/09/09]
52. Patterson PH. Maternal infection: window on neuroimmune interactions in fetal brain development and mental illness. *Curr Opin Neurobiol* 2002;12(1):115-8. doi: 10.1016/s0959-4388(02)00299-4 [published Online First: 2002/02/28]
53. Bland ST, Beckley JT, Young S, et al. Enduring consequences of early-life infection on glial and neural cell genesis within cognitive regions of the brain. *Brain Behav Immun* 2010;24(3):329-38. doi: 10.1016/j.bbi.2009.09.012 [published Online First: 2009/09/29]
54. Money KM, Barke TL, Serezani A, et al. Gestational diabetes exacerbates maternal immune activation effects in the developing brain. *Mol Psychiatry* 2018;23(9):1920-28. doi: 10.1038/mp.2017.191 [published Online First: 2017/09/28]
55. Bilbo SD, Schwarz JM. Early-life programming of later-life brain and behavior: a critical role for the immune system. *Front Behav Neurosci* 2009;3:14. doi: 10.3389/neuro.08.014.2009 [published Online First: 2009/09/10]
56. Buss C, Davis EP, Shahbaba B, et al. Maternal cortisol over the course of pregnancy and subsequent child amygdala and hippocampus volumes and affective problems. *P Natl Acad Sci USA* 2012;109(20):E1312-E19. doi: 10.1073/pnas.1201295109
57. Yehuda R, Lehrner A. Intergenerational transmission of trauma effects: putative role of epigenetic mechanisms. *World Psychiatry* 2018;17(3):243-57. doi: 10.1002/wps.20568 [published Online First: 2018/09/08]
58. Cooper PJ, Tomlinson M, Swartz L, et al. Post-partum depression and the mother-infant relationship in a South African peri-urban settlement. *Br J Psychiatry* 1999;175:554-8. doi: 10.1192/bjp.175.6.554 [published Online First: 2000/05/02]
59. Tomlinson M, Cooper P, Murray L. The mother-infant relationship and infant attachment in a South African peri-urban settlement. *Child Dev* 2005;76(5):1044-54. doi: 10.1111/j.1467-8624.2005.00896.x [published Online First: 2005/09/10]

For peer review only

Contributors: WPF and HJO acquired funding for and designed the work. LCS, AS, NHB, ML, YR, PES, HJO, and WPF conceptualized the work. MP, PES, CDP, and HJO acquired the data. LCS, NHB, and WPF led in data collection oversight and quality control. AS, LCS, and YR

For peer review only - http://bmjopen.bmj.com/site/about/guidelines.xhtml

analyzed the data. All authors contributed to the interpretation of data and drafting the work and revising it critically for important intellectual content. All authors approved the final manuscript and agree to be accountable for all aspects of the work.

Acknowledgements: We would like to acknowledge Lucy Brink, J. David Nugent, and Daianna Rodriguez for their database and administrative support. We would also like to thank all of the families who participated in this research study for making this work possible.

Ethics statements

Patient consent for publication

Not applicable.

Ethics approval

Ethics approval was received from the Institutional Review Boards and ethics review committees at Stellenbosch University (N16-08-101 and N06-10-210) and the New York State Psychiatric Institute (5338). All participants provided informed written consent at both time points (prenatal and postnatal) before inclusion in the study.

For peer review only

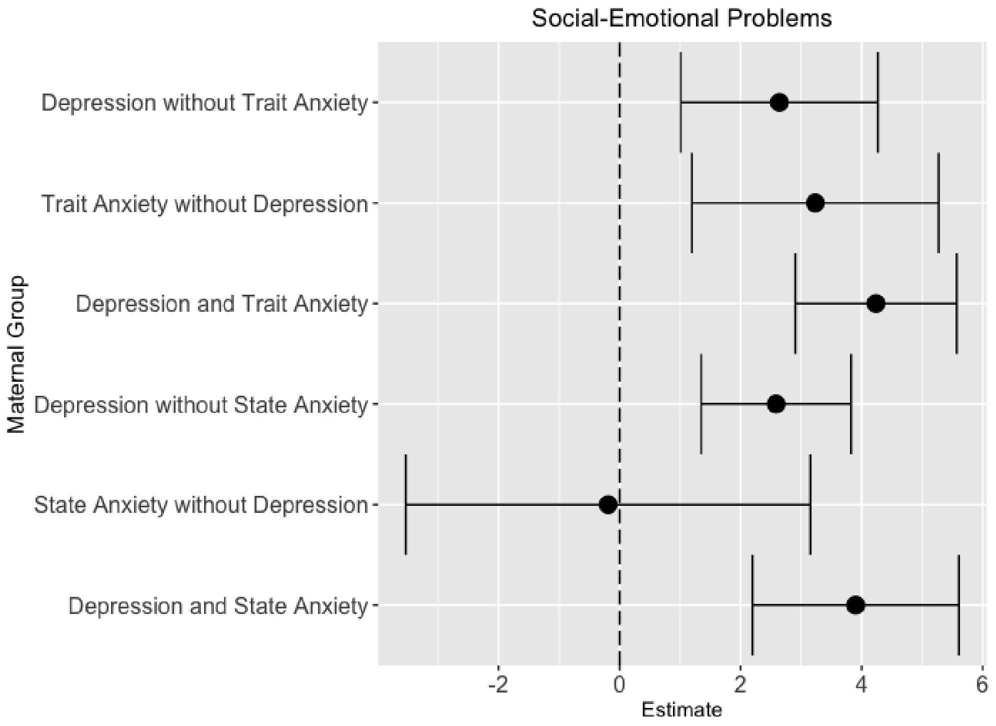

Figure 1. Association between prenatal maternal depression, prenatal maternal anxiety, and child social-emotional problems measured by the BITSEA. Each line plot depicts the mean difference and their confidence interval for child social-emotional problems (x-axis) for each prenatal maternal mental health group (y-axis). Either no prenatal maternal depression and state anxiety or no prenatal maternal depression and trait anxiety were the reference groups. Models were adjusted for sex, gestational age at birth, age at follow-up, prenatal maternal alcohol use, prenatal maternal tobacco use, maternal employment status at delivery, maternal educational attainment at delivery, parity, and the household crowding index.

247x181mm (72 x 72 DPI)

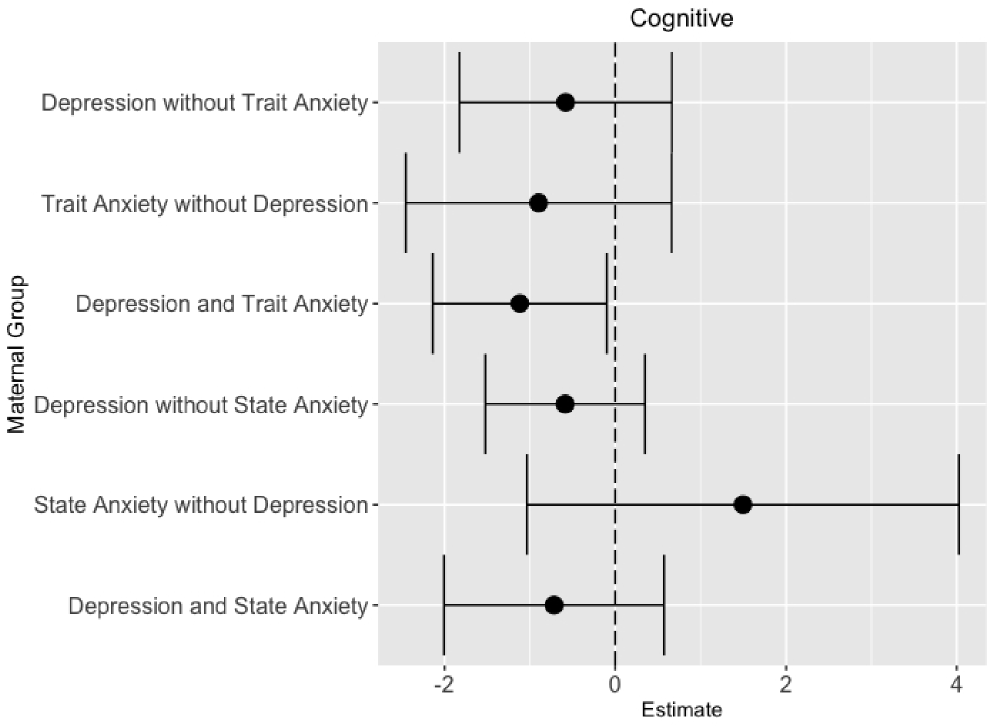

Figure 2. . Association between prenatal maternal depression, prenatal maternal anxiety, and child cognitive development measured by the BSID-III Screening Test. Each line plot depicts the mean difference and their confidence interval for child cognitive development (x-axis) for each prenatal maternal mental health group (y-axis). Either no prenatal maternal depression and state anxiety or no prenatal maternal depression and trait anxiety were the reference groups. Models were adjusted for sex, gestational age at birth, age at follow-up, prenatal maternal alcohol use, prenatal maternal tobacco use, maternal employment status at delivery, maternal educational attainment at delivery, parity, and the household crowding index.

247x181mm (72 x 72 DPI)

STROBE Statement—Checklist of items that should be included in reports of ***cohort studies***

	Item No	Recommendation	Page No
**Title and abstract**	1	(*a*) Indicate the study's design with a commonly used term in the title or the abstract	2
		(*b*) Provide in the abstract an informative and balanced summary of what was done and what was found	3, 4
**Introduction**			
Background/rationale	2	Explain the scientific background and rationale for the investigation being reported	5, 6
Objectives	3	State specific objectives, including any prespecified hypotheses	6
**Methods**			
Study design	4	Present key elements of study design early in the paper	7 - 9
Setting	5	Describe the setting, locations, and relevant dates, including periods of recruitment, exposure, follow-up, and data collection	7 - 9
Participants	6	(*a*) Give the eligibility criteria, and the sources and methods of selection of participants. Describe methods of follow-up	7 - 9
		(*b*) For matched studies, give matching criteria and number of exposed and unexposed	N/A
Variables	7	Clearly define all outcomes, exposures, predictors, potential confounders, and effect modifiers. Give diagnostic criteria, if applicable	9 - 10
Data sources/ measurement	8*	For each variable of interest, give sources of data and details of methods of assessment (measurement). Describe comparability of assessment methods if there is more than one group	7 - 10
Bias	9	Describe any efforts to address potential sources of bias	10
Study size	10	Explain how the study size was arrived at	11
Quantitative variables	11	Explain how quantitative variables were handled in the analyses. If applicable, describe which groupings were chosen and why	9 - 10
Statistical methods	12	(*a*) Describe all statistical methods, including those used to control for confounding	9 - 10
		(*b*) Describe any methods used to examine subgroups and interactions	9 - 10
		(*c*) Explain how missing data were addressed	10
		(*d*) If applicable, explain how loss to follow-up was addressed	10
		(*e*) Describe any sensitivity analyses	N/A
**Results**			
Participants	13*	(a) Report numbers of individuals at each stage of study—eg numbers potentially eligible, examined for eligibility, confirmed eligible, included in the study, completing follow-up, and analysed	11
		(b) Give reasons for non-participation at each stage	11
		(c) Consider use of a flow diagram	
Descriptive data	14*	(a) Give characteristics of study participants (eg demographic, clinical, social) and information on exposures and potential confounders	9, 11 - 13
		(b) Indicate number of participants with missing data for each variable of interest	10
		(c) Summarise follow-up time (eg, average and total amount)	
Outcome data	15*	Report numbers of outcome events or summary measures over time	11

For peer review only - http://bmjopen.bmj.com/site/about/guidelines.xhtml

Main results	16	(*a*) Give unadjusted estimates and, if applicable, confounder-adjusted estimates and their precision (eg, 95% confidence interval). Make clear which confounders were adjusted for and why they were included	11 - 13
		(*b*) Report category boundaries when continuous variables were categorized	N/A
		(*c*) If relevant, consider translating estimates of relative risk into absolute risk for a meaningful time period	
Other analyses	17	Report other analyses done—eg analyses of subgroups and interactions, and sensitivity analyses	11 - 13
**Discussion**			
Key results	18	Summarise key results with reference to study objectives	14
Limitations	19	Discuss limitations of the study, taking into account sources of potential bias or imprecision. Discuss both direction and magnitude of any potential bias	17
Interpretation	20	Give a cautious overall interpretation of results considering objectives, limitations, multiplicity of analyses, results from similar studies, and other relevant evidence	17
Generalisability	21	Discuss the generalisability (external validity) of the study results	17
**Other information**			
Funding	22	Give the source of funding and the role of the funders for the present study and, if applicable, for the original study on which the present article is based	1

*Give information separately for exposed and unexposed groups.

Note: An Explanation and Elaboration article discusses each checklist item and gives methodological background and published examples of transparent reporting. The STROBE checklist is best used in conjunction with this article (freely available on the Web sites of PLoS Medicine at http://www.plosmedicine.org/, Annals of Internal Medicine at http://www.annals.org/, and Epidemiology at http://www.epidem.com/). Information on the STROBE Initiative is available at http://www.strobe-statement.org.

For peer review only

Association of Maternal Depression and Anxiety with Toddler Social-Emotional and Cognitive Development in South Africa: A prospective cohort study

Journal:	*BMJ Open*
Manuscript ID	bmjopen-2021-058135.R2
Article Type:	Original research
Date Submitted by the Author:	17-Mar-2022
Complete List of Authors:	Shuffrey, Lauren; Columbia University Irving Medical Center, Psychiatry; New York State Psychiatric Institute, Division of Developmental Neuroscience<br>Sania, Ayesha; Columbia University Irving Medical Center, Psychiatry; New York State Psychiatric Institute, Division of Developmental Neuroscience<br>Brito, Natalie; New York University, Department of Applied Psychology<br>Potter, Mandy; Stellenbosch University Faculty of Medicine and Health Sciences, Obstetrics and Gynaecology<br>Springer, Priscilla; Stellenbosch University, Paediatrics and Child Health<br>Lucchini, Maristella ; Columbia University Irving Medical Center, Psychiatry; New York State Psychiatric Institute, Neuroscience<br>Rayport, Yael; Columbia University Irving Medical Center, Department of Psychiatry; New York State Psychiatric Institute, Neuroscience<br>Du Plessis, Carlie; Stellenbosch University Faculty of Medicine and Health Sciences, Department of Obstetrics and Gynaecology<br>Odendaal, H. J.; Stellenbosch University Faculty of Medicine and Health Sciences, Obstetrics & Gynaecology<br>Fifer, William; Columbia University Irving Medical Center, Psychiatry; New York State Psychiatric Institute, Neuroscience
**Primary Subject Heading**:	Global health
Secondary Subject Heading:	Mental health, Paediatrics
Keywords:	Community child health < PAEDIATRICS, Depression & mood disorders < PSYCHIATRY, Anxiety disorders < PSYCHIATRY, Child & adolescent psychiatry < PSYCHIATRY

I, the Submitting Author has the right to grant and does grant on behalf of all authors of the Work (as defined in the below author licence), an exclusive licence and/or a non-exclusive licence for contributions from authors who are: i) UK Crown employees; ii) where BMJ has agreed a CC-BY licence shall apply, and/or iii) in accordance with the terms applicable for US Federal Government officers or employees acting as part of their official duties; on a worldwide, perpetual, irrevocable, royalty-free basis to BMJ Publishing Group Ltd ("BMJ") its licensees and where the relevant Journal is co-owned by BMJ to the co-owners of the Journal, to publish the Work in this journal and any other BMJ products and to exploit all rights, as set out in our licence.

The Submitting Author accepts and understands that any supply made under these terms is made by BMJ to the Submitting Author unless you are acting as an employee on behalf of your employer or a postgraduate student of an affiliated institution which is paying any applicable article publishing charge ("APC") for Open Access articles. Where the Submitting Author wishes to make the Work available on an Open Access basis (and intends to pay the relevant APC), the terms of reuse of such Open Access shall be governed by a Creative Commons licence – details of these licences and which Creative Commons licence will apply to this Work are set out in our licence referred to above.

Other than as permitted in any relevant BMJ Author's Self Archiving Policies, I confirm this Work has not been accepted for publication elsewhere, is not being considered for publication elsewhere and does not duplicate material already published. I confirm all authors consent to publication of this Work and authorise the granting of this licence.

For peer review only

Title: Association of Maternal Depression and Anxiety with Toddler Social-Emotional and Cognitive Development in South Africa: A prospective cohort study

Lauren C. Shuffrey*[1,2], Ayesha Sania*[1,2], Natalie H. Brito[3], Mandy Potter[4], Priscilla E. Springer[5], Maristella Lucchini[1,2], Yael K. Rayport[1,2], Carlie Du Plessis[4], Hein J. Odendaal[4], William P. Fifer[1,2,6]

[1] Department of Psychiatry, Columbia University Irving Medical Center, New York, NY 10032 USA
[2] Division of Developmental Neuroscience, New York State Psychiatric Institute, New York, NY 10032 USA
[3] Department of Applied Psychology, New York University, New York, NY, 10003 USA
[4] Department of Obstetrics and Gynaecology, Faculty of Medicine and Health Science, Stellenbosch University, Cape Town, Western Cape, South Africa 7530
[5] Department of Paediatrics and Child Health, Stellenbosch University, Cape Town, Western Cape, South Africa 7530
[6] Department of Pediatrics, Columbia University Irving Medical Center, New York, NY 10032 USA
*** denotes shared authorship**

Corresponding Author:
Lauren C. Shuffrey, PhD
Columbia University Medical Center
New York State Psychiatric Institute
1051 Riverside Drive, Unit 40
New York, New York 10032
lcg2129@cumc.columbia.edu

Financial Support: This research was supported by the Bill and Melinda Gates Foundation (WPF) and grants U01HD055154, U01HD045935, U01HD055155, and U01HD045991 issued by the *Eunice Kennedy Shriver* National Institute of Child Health and Human Development (NICHD) and grant U01AA016501 issued by the National Institute on Alcohol Abuse and Alcoholism (NIAAA). Dr. Lauren Shuffrey is supported by K99HD103910 issued by the Eunice Kennedy Shriver National Institute Of Child Health & Human Development. Dr. Ayesha Sania is supported by UH3OD023279-05S1, re-entry supplement from Office of the Director, NIH, and Office of Research on Women Health (ORWH). The content is solely the responsibility of the authors and does not necessarily represent the official views of the National Institutes of Health.

Competing Interests: The authors have no competing interests to declare.

Data availability: Data are available upon reasonable request.

Patient and Public Involvement: Patients or the public were not involved in the design, or conduct, or reporting, or dissemination plans of our research

For peer review only - http://bmjopen.bmj.com/site/about/guidelines.xhtml

Abstract:

Objective: A robust literature has identified associations between prenatal maternal depression and adverse child social-emotional and cognitive outcomes. The majority of prior research is from high-income countries despite increased reporting of perinatal depression in low and middle income countries (LMICs). Additionally, despite the comorbidity between depression and anxiety, few prior studies have examined their joint impact on child neurodevelopment. The objective of the current analysis was to examine associations between prenatal maternal depression and anxiety with child social-emotional and cognitive development in a cohort from the Western Cape Province of South Africa.

Design: Prenatal maternal depression and anxiety were measured using the Edinburgh Postnatal Depression Scale (EPDS) and the State-Trait Anxiety Scale (STAI) at 20 – 24 weeks' gestation. Child neurobehavior was assessed at age 3 using the Brief Infant Toddler Social Emotional Assessment (BITSEA) and the Bayley Scales of Infant Development Screening Test (BSID-III ST). We used linear regression models to examine the independent and joint association between prenatal maternal depression, anxiety, and child developmental outcomes.

Results: Participants consisted of 600 maternal-infant dyads (274 females; gestational age at birth: 38.89 weeks ± 2.03). Children born to mothers with both prenatal depression and trait anxiety had higher social-emotional problems (mean difference: 4.66; 95% CI 3.43, 5.90) compared to children born to mothers with no prenatal depression or trait anxiety, each condition alone, or compared to mothers with depression and state anxiety. Additionally, children born to mothers with prenatal maternal depression and trait anxiety had the greatest reduction in mean cognitive scores on the BSID-III ST (mean difference: -1.04; 95% CI -1.99, -0.08).

Conclusions: The observed association between comorbid prenatal maternal depression and chronic anxiety with subsequent child social-emotional and cognitive development underscores the need for targeting mental health support in perinatal women in LMICs to improve long-term child neurobehavioral outcomes.

For peer review only

For peer review only - http://bmjopen.bmj.com/site/about/guidelines.xhtml

Strengths and limitations of this study

- The current study included a prospective evaluation of maternal depression and anxiety symptoms during pregnancy and prospective assessment of cognitive and social-emotional outcomes within the same cohort in a large sample of South African mother-children pairs.
- Limitations include a lack of data on maternal mental health assessments postnatally and mother-child dyadic measures, which are potential mediators of the relationship between prenatal maternal depression, prenatal maternal anxiety, and child developmental outcomes.
- This study addresses a significant gap in the literature in research examining the impact of prenatal maternal psychological health on child neurobehavioral development in resource-poor communities.

Keywords: prenatal depression, prenatal anxiety; social-emotional development; cognitive development

For peer review only

Introduction

Decades of research on the early origins of behavior has promoted the concept that the prenatal environment has a profound impact on one's risk for the development of neurodevelopmental or psychiatric disorders[1 2]. Several prior studies have identified associations between prenatal maternal depression and increased risk for social-emotional problems and decrements in cognitive development. However, the majority of prior research in this domain is from high-income countries (HICs), despite increased reporting of perinatal depression in low and middle income countries (LMICs) including South Africa where an estimated 35% women report prenatal depression[3 4]. Additionally, despite the comorbidity between depression and anxiety, few prior studies have examined their joint impact on child neurodevelopment.

Several studies in HICs have identified associations between prenatal maternal depression, anxiety, and offspring behavioral, social-emotional, and cognitive development[5]. Specifically, a meta-analysis demonstrated adverse effects of prenatal maternal depression and anxiety on child social-emotional problems, with odds ratios of 1.79 and 1.50 respectively[6]. Prenatal maternal depression and anxiety are also associated with cognitive and language deficits[7 8], delayed motor development[8], emotional and behavior dysregulation[9-11], inattention and hyperactivity[12-14], and difficult temperament[15]. A more recent meta-analysis not only confirmed prior reports, but also found that the effects of perinatal maternal depression extend beyond infancy through adolescence[16].

The developmental origins of health and disease (DOHaD) model posits that maternal psychological distress during pregnancy (e.g. perceived stress, depression, anxiety, post-traumatic stress) may result in changes in hypothalamic pituitary adrenal (HPA) axis function and upregulation of inflammatory processes with downstream effects on offspring perinatal brain

For peer review only - http://bmjopen.bmj.com/site/about/guidelines.xhtml

development and behavior[17]. Prior research suggests comorbid prenatal maternal depression and anxiety may be associated with the greatest increases in maternal HPA-axis activity and differential changes in immunologic activity. Specifically, comorbid prenatal maternal depression and anxiety have been associated with a greater increase in salivary cortisol levels[18], TH1 secreted cytokines, TH2 secreted cytokines, and TH17 secreted cytokines[19] compared to either condition alone. Other research suggests cytokine profiles may differ between individuals with prenatal maternal depression and anxiety[19-21].

Risk factors may be exacerbated in LMICs such as South Africa, where both poverty and perinatal mental health disorders are highly prevalent[22 23]. Specifically, in South Africa maternal mood disorders have been linked to structural and community stressors associated with markers of poverty including less than a high school education, lack of social support, alcohol use, family stress, food insecurity, lack of partner involvement, and intimate partner violence[24-28]. There are few prior studies that have examined the impact of prenatal maternal mental health on offspring social-emotional or cognitive development in South Africa. While the few prior studies reported significant harmful effects of prenatal maternal stress or perinatal maternal depression, there is still a significant gap in the literature in research examining the impact of prenatal maternal psychological health on child neurobehavioral development in resource-poor communities[24 29 30]. Additionally, to our knowledge no prior South African studies have examined the joint effect of prenatal maternal depression and anxiety on child neurobehavioral outcomes. Research examining the long-term impact of prenatal maternal depression and anxiety on child behavioral and cognitive outcomes is critical for providing justification to local public health services for targeting mental health support in perinatal women from underserved communities.

For peer review only - http://bmjopen.bmj.com/site/about/guidelines.xhtml

The objective of the current analysis was to determine if prenatal maternal depression and state or trait anxiety were associated with child social-emotional problems or cognitive development at approximately three years of age in a South African cohort from the Western Cape.

Materials and Methods

Participants. Participants were a subset of infants with available outcome data at age 3 enrolled in the Safe Passage Study conducted by the Prenatal Alcohol and SIDS and Stillbirth (PASS) Network, a multi-center study investigating the role of prenatal exposure in risk for sudden infant death syndrome (SIDS), stillbirth, and fetal alcohol spectrum disorders. Eligibility criteria for the Safe Passage study included the ability to provide informed consent in English or Afrikaans, 16 years of age or older at the time of consent, and a gestational age between 6 weeks and 40 weeks at the time of consent based on estimated delivery date[31]. Exclusion Criteria for prenatal maternal enrollment into the Safe Passage study included planned therapeutic abortion, moving out of the catchment area prior to estimated date of delivery, and clinical judgment. Informed consent was obtained for the Safe Passage study and from a parent or legal guardian of each participant for developmental follow-up assessments. Ethical approval was obtained for both time points from the Health Research Ethics Committee of Stellenbosch University and the New York State Psychiatric Institute.

Maternal Assessments.

Maternal-infant chart abstraction, demographic, and socioeconomic measures. Maternal-infant medical charts were abstracted to obtain maternal age at delivery, gestational age at birth,

For peer review only - http://bmjopen.bmj.com/site/about/guidelines.xhtml

mode of delivery, and the infant's biological sex. Measures to collect prenatal alcohol, tobacco, and recreational drug exposure have been previously described[31 32]. Prenatal maternal alcohol and tobacco use behaviors were previously characterized using cluster analysis[33 34]. Through study specific case report forms, participants indicated demographic and socioeconomic information including race, maternal educational attainment, household crowding (persons per room in household), access to running water inside the house, prenatal care during pregnancy, and marital status.

Self-reported depression and anxiety measures. Information regarding maternal mental health during pregnancy was obtained at 20 – 24 weeks' gestation. Depressive symptoms were measured using the Edinburgh Postnatal Depression Scale (EPDS), a depression screening tool developed to specifically assess depressive symptoms in perinatal women where higher scores indicate more severe depressive symptoms[35 36]. The EPDS is widely used and has been validated in English and Afrikaans in South Africa[35 37]. Prior studies have used a cut-off score of ≥ 12 or ≥ 13 to be indicative of major depression within perinatal women living in South Africa[35 37]. Maternal anxiety symptoms were measured using the State-Trait Anxiety Inventory (STAI)[38], an anxiety screening tool to distinguish anxiety symptoms from depressive symptoms which has also been validated in both languages[39]. The STAI has two subscales, state anxiety which reflects the participant's current state of anxiety when completing the questionnaires and trait anxiety, which is thought to be consistent across time and reflect personality traits. In HICs, the STAI has a cut-off score of ≥ 40 on both the state anxiety and trait anxiety subscales to indicate a threshold for clinical levels of anxiety. Based on these prior studies, we used a cutoff of ≥ 13 to indicate maternal depression, a cutoff of > 40 on the STAI-state subscale to indicate state anxiety, and a

For peer review only - http://bmjopen.bmj.com/site/about/guidelines.xhtml

cutoff of > 40 on the STAI-trait subscale to indicate trait anxiety.

Toddler Developmental Assessments.

Bayley Scales of Infant Development III Screening Test. The Bayley Scales of Infant Development III Screening Test (BSID-III ST) were designed as a rapid assessment of cognitive, language, and motor functioning in infants and young children in order to determine if a child's development is within normal limits and identify risk for developmental delay. The BSID-III ST has high test-retest reliability: Cognitive (0.85), Receptive Language (0.88), Expressive Language (0.88), Fine Motor (0.82), and Gross Motor (0.86). Although the BSID-III ST does not identify degree of impairment, the cut-off points indicate whether a child shows competence in age-appropriate tasks, evidence of emerging age-appropriate skills, and evidence of being at risk for developmental delay. The BSID has been validated and widely used throughout South Africa[40 41].

Brief Infant Toddler Social Emotional Assessment (BITSEA). The Brief Infant-Toddler Social and Emotional Assessment (BITSEA) is a 42-item parental report measure of social-emotional development, behavioral problems, and delays in competence[42]. Domains assessed within the BITSEA include: externalizing (activity/impulsivity, aggression/defiance, peer aggression), internalizing (depression/withdrawal, anxiety, separation distress, inhibition to novelty), dysregulation (sleep, negative emotionality, eating, sensory sensitivity), and competence (compliance, attention, imitation/play, mastery motivation, empathy, and pro-social peer relations)[42]. Findings from the BITSEA validation study provide preliminary support for the BITSEA as a reliable and valid brief screener for infant-toddler social-emotional and behavioral problems in addition to delays in competence[43]. When used in socioeconomically and ethnically

For peer review only - http://bmjopen.bmj.com/site/about/guidelines.xhtml

diverse community-based populations, the BITSEA demonstrated excellent test-retest reliability and good inter-rater agreement between parents[42].

Statistical Analyses. Using multiple linear regression models, we estimated independent and joint effects of maternal depression and state and trait anxiety on social-emotional problem, social emotional competence, and cognitive development scores. Two, separate four-level categorical prenatal maternal mental health variables were created to assess the impact of prenatal maternal depression, trait anxiety, and state anxiety. We created a prenatal maternal depression and trait anxiety variable with four categories: (1) *No Prenatal Depression or Trait Anxiety* (n=199; 33.17%, Reference Category), (2) *Prenatal Depression Only* (106; 17.67%), (3) *Prenatal Trait Anxiety Only* (n=68; 11.33%) and (4) *Prenatal Maternal Depression and Trait Anxiety* (n=227; 37.83%) (Table 1). In separate models we additionally examined the independent and joint effects of prenatal maternal depression and state anxiety. We created a prenatal maternal depression and state anxiety variable with four categories: (1) *No Prenatal Depression or State Anxiety* (n=248; 41.33%; Reference Category), (2) *Prenatal Depression Only* (n=237; 39.50%), (3) *Prenatal State Anxiety Only* (n=19; 3.17%) and (4) *Prenatal Maternal Depression and State Anxiety* (n=96; 16%) (Table 1). For each regression model, either *No Prenatal Maternal Depression or Trait Anxiety* or *No Prenatal Maternal Depression and State Anxiety* was set as the reference category. Minimally adjusted models included sex, gestational age at birth, and age at follow up as covariates. Fully adjusted models additionally controlled for prenatal maternal alcohol use, prenatal maternal tobacco use, maternal employment status at delivery, maternal educational attainment at delivery, parity, and the household crowding index. We used missing indicator methods and median imputation to

For peer review only - http://bmjopen.bmj.com/site/about/guidelines.xhtml

account for missing categorial and continuous covariate data, respectively (described in Table 1). All analyses were performed in SAS software version 9.4 (SAS Institute, Cary NC).

Results

Maternal and Child Demographic Characteristics. The participants included in the present analysis consisted of mothers and their infant born between April 2014 and August 2015 from the Western Cape Province of South Africa who participated in a follow-up study to examine social-emotional development and cognitive development at approximately three years of age. A total of n=18 mother-infant dyads were excluded due to missing maternal prenatal mental health data. The final sample consisted of 600 maternal-infant dyads (274 females; gestational age at birth: 38.89 weeks ± 2.03) (Table 1).

Child Social-Emotional Development. Based on the BITSEA problem scale percentile rank score of 26 or higher, 51% of children (306/600) were classified as having a "possible problem". Based on the BITSEA competence scale percentile rank score of 15 of lower, 5% (30/600) of children were classified in the "possible deficit/delay range" for social competencies (Table 2). There were no significant sex differences in social-emotional problems on the BITSEA, however girls had significantly higher social-emotional competence compared to boys (mean difference: 0.38, CI: 0.05, 0.71, p = 0.03)

Association between Prenatal Maternal Depression, Trait Anxiety, and Child Social-Emotional Development. Compared to children born to mothers with no prenatal depression or trait anxiety, children born to mothers with prenatal depression and trait anxiety had higher social-emotional problems (mean difference: 4.66; 95% CI 3.43, 5.90), followed by prenatal maternal trait anxiety only (mean difference: 3.87; 95% CI 2.07, 5.66), and finally prenatal maternal depression only (mean difference: 2.76; 95% CI 1.23, 4.29) in minimally adjusted

For peer review only - http://bmjopen.bmj.com/site/about/guidelines.xhtml

For peer review only

models. These associations remained significant in fully adjusted models where similarly comorbid prenatal maternal depression and trait anxiety group was associated with the highest child social-emotional problems (mean difference: 4.33; 95% CI 2.90, 5.67), followed by prenatal maternal trait anxiety only (mean difference: 3.23; 95% CI 1.19, 5.27), with the smallest mean difference for prenatal maternal depression only group (mean difference: 2.64; 95% CI 1.02, 4.27) as compared to the no prenatal depression or trait anxiety group (Figure 1). Additional significant predictors in the multivariate models were parity of 3 or greater which was associated with lower social-emotional problems (mean difference: -3.02, 95% CI -4.63, -1.40) and low continuous smoking during pregnancy which was associated with higher social-emotional problems (mean difference: 1.39, 95% CI 0.039, 2.74). There were no significant associations between prenatal maternal depression, trait anxiety, and child social-emotional competence.

Association between Prenatal Maternal Depression, State Anxiety, and Child Social-Emotional Development. Compared to children born to mothers with no prenatal depression or state anxiety, children born to mothers with comorbid prenatal depression and state anxiety had higher social-emotional problems (mean difference: 4.29; 95% CI 2.73, 5.84). Children born to mothers with prenatal depression only also had higher social-emotional problems compared to mothers with no prenatal depression or state anxiety (mean difference: 2.71: 95% CI 1.51, 3.88). These associations remained significant in fully adjusted models (prenatal depression and state anxiety: 3.90 mean increase (95% CI 2.19, 5.60); prenatal depression only: 2.58 mean increase (95% CI 1.34, 3.82)) (Figure 1). Additional significant predictors in the multivariate models were parity of 3 or greater which was associated with lower social-emotional problems (mean difference: -3.22, 95% CI -4.86, -1.58), low continuous smoking during pregnancy which was

For peer review only - http://bmjopen.bmj.com/site/about/guidelines.xhtml

associated with higher social-emotional problems (mean difference: 1.44; 95% CI 0.075, 2.81), and finally less than a high school education (some primary school only) which was associated with higher social-emotional problems (mean difference: 3.47; 95% CI 0.248, 6.70). However, there was no significant association between prenatal state anxiety only and child social-emotional problems on the BITSEA. There were also no significant associations between prenatal maternal depression, state anxiety, and social-emotional competence.

Child Cognitive, Language, and Motor Development. Based on normative cognitive cutoff scores defined by the BSID-III ST, 4% of children (24/600) were classified as at-risk (Table 2). Risk classification percentages were similar across all subdomains, expressive language: 4% , receptive language: 6%, gross motor: 4%, and fine motor: 3%. There were no significant sex differences in child cognitive scores.

Association between Prenatal Maternal Depression, Trait Anxiety, and Child Cognitive, Language, and Motor Development. Compared to children born to mothers with no prenatal depression or trait anxiety, children born to mothers with comorbid prenatal depression and trait anxiety had lower cognitive scores on the BSID-III ST (mean difference: -1.04; 95% CI -1.99, -0.08). Results remained significant in the fully adjusted model (mean difference: -1.11; 95% CI -2.13, -0.09) (Figure 2) and in posthoc analyses where we additionally controlled for language of administration for the BSID-III ST (mean difference: -0.51, 95% CI -0.99, -0.042). Children who were assessed on the BSID-III ST in Afrikaans (mean difference: -1.00, 95% CI -1.48, -0.52) or who were assessed in mixed English and Afrikaans (mean difference: -1.30, 95% CI -2.07, -0.54) has significantly lower cognitive scores compared to children assessed in English. Low continuous prenatal maternal alcohol use was also associated with lower cognitive scores (mean difference: -1.30; 95% CI -2.36, -0.24).

For peer review only - http://bmjopen.bmj.com/site/about/guidelines.xhtml

Association between Prenatal Maternal Depression, State Anxiety, and Child Cognitive, Language, and Motor Development. Compared to children born to mothers with neither prenatal depression nor trait anxiety, there was no significant association between cognitive scores for children born to mothers with prenatal depression only, prenatal trait anxiety only, or combined prenatal depression and trait anxiety in either the minimally or fully adjusted models.

Discussion

In summary, we found the greatest increase in child social-emotional problems in children born to women with comorbid prenatal depression and trait anxiety compared to women with no prenatal depression or trait anxiety, prenatal depression alone, or prenatal trait anxiety alone. We additionally found a significant association between comorbid prenatal maternal depression and state anxiety on higher child social-emotional problems; however, we found no association between prenatal maternal state anxiety in the absence of prenatal maternal depression on child social-emotional problems. Finally, we reported children born to mothers with prenatal maternal depression and trait anxiety had lower cognitive scores on the BSID-III ST, but we did not find an association between prenatal maternal depression alone on cognitive outcomes at 3-years of age.

Our finding linking prenatal maternal depression and anxiety to social-emotional risk parallels two recent South African studies linking increased prenatal maternal stressors to higher behavioral problems in children with an odds ratio of 2.52, but not before 4 years of age[29] and increased perinatal depression to aggressive behaviors at 60 months of age[24]. Our findings are also largely consistent with a recent meta-analysis of studies predominately conducted in HICs, which found prenatal maternal depression and anxiety were associated with higher social-

For peer review only - http://bmjopen.bmj.com/site/about/guidelines.xhtml

emotional problems in offspring with larger effect sizes for prenatal maternal depression (OR 1.79; 95% CI 1.61 - 1.99) compared to prenatal maternal anxiety (OR 1.50, 95% CI, 1.36 - 1.64)[6]. We found the greatest increase in child social-emotional problems in children born to women with comorbid prenatal depression and trait anxiety and no effect of prenatal maternal state anxiety in the absence of prenatal maternal depression on child social-emotional problems. Our results are suggestive that chronic anxiety measured via trait anxiety may be more predictive of child social-emotional outcomes than a single measurement of concurrent anxiety during pregnancy.

A prior meta-analysis[6] also found stronger effects of prenatal maternal depression and anxiety on child social-emotional outcomes when sociodemographic risk factors such as low-income, lower levels of parental education, or single family households, were highest[6]. Similarly, we found that lower levels of maternal education, low continuous tobacco use during pregnancy, and low continuous alcohol use during pregnancy were associated with higher social-emotional problems in children at three years of age. Intriguingly, maternal parity of 3 or greater was protective and associated with lower social-emotional problems which may be due to reduced depression and anxiety levels in women with higher parity, changes in the perception of their child's behavior due to having multiple children, or additional social opportunities during sibling interactions[44 45].

The Drakenstein Child Health Study based in the Western Cape found approximately 50% of the overall sample (369/731) were categorized as having cognitive delay at two years of age based on cutoffs defined by United States (US) normative data[30]. Better cognitive outcomes were associated with higher maternal education, older child age, a primigravid mother, and higher socioeconomic status whereas prenatal maternal depression was associated with a 1.03

For peer review only - http://bmjopen.bmj.com/site/about/guidelines.xhtml

SD (95% CI -1.94 to -0.12) reduction in cognitive scores on the Bayley Scales of Infant and Toddler Development at two years of age[30]. Similar to the Drakenstein Child Health Study[30], we found children born to mothers with prenatal maternal depression and trait anxiety had lower cognitive scores on the BSID-III ST compared to the no prenatal maternal depression or trait anxiety group. In contrast, we did not find an association between prenatal maternal depression alone on cognitive outcomes at 3-years of age. However, since we did not utilize the full BSID-III and only administered the BSID-III ST, it is difficult to directly compare our results to The Drakenstein Child Health study findings. Moreover, both studies relied on US normative data to define cutoff scores.

More recently, a large home-visiting intervention study based in Cape Town examined the effect of prenatal maternal depression only, postnatal maternal depression only (birth – 60 months), or recurrent pre- and postnatal maternal depression. This study also accounted for several other risk factors including intimate partner violence, HIV status, and alcohol use on child social behaviors, language skills, and cognitive development. No associations were found between maternal depression at any time point with children's language or cognitive development at 36 or 60 months of age[24]. However, children of never depressed mothers had lower aggressive behaviors on the Child Behavior Checklist at 60 months of age than children of mothers with postnatal depression only or perinatal depression[24].

There are several biological mechanisms which can explain prior studies and our current findings linking prenatal maternal depression and anxiety with child social-emotional behaviors and cognitive development such as increased prenatal maternal inflammation, increased cortisol production, and/or epigenetic changes[46-55]. Pregnancy is associated with changes in inflammatory processes and increased placental cortisol production with reduced maternal HPA axis sensitivity

For peer review only

to stress[46 47]. However, prenatal maternal depression may upregulate inflammatory processes and/or cortisol production. Additionally, prior research found women with comorbid depression and anxiety have the greatest increases in salivary cortisol levels[18]. Maternal cortisol can cross the placenta resulting in increased inflammation[46 47] and/or affect the developmental of limbic regions, which are associated with social and emotional processes[56]. In animal models, increased proinflammatory proteins have been associated with widespread changes in perinatal brain development such as with volume reductions in gray and white matter, decreased density of GABAergic neurons, reduced synaptic pruning, and network dysfunction with potential downstream effects on neurobehavioral development[48-55]. Other studies examining the intergenerational transmission of trauma have demonstrated transgenerational epigenetic changes in animal models[57]. It is also possible comorbid maternal depression and trait anxiety may indicate a specific phenotype that is genetically transmitted to the next generation resulting in psychosocial sequelae in offspring. Taken together, prior research suggests multiple overlapping pathways by which prenatal maternal mood can affect offspring brain-behavioral development.

While we report an association of prenatal maternal depression and anxiety with child social-emotional and cognitive development from a prospective cohort study with a fairly large sample of participants, there are several methodological and contextual limitations within the current study that are worth noting. First, it is important to note that the reliance on maternal-report measures to characterize child social-emotional development is a limitation in the majority of research to date, including the present study. The reliance on maternal reporting of child social-emotional development may be influenced by factors such as maternal mood or education. An additional limitation was the use of the BSID-III screening test in the current study to measure cognitive development, which is based on US normative data. Future studies should

For peer review only

consider objective measures of child social-emotional development through observational or behavioral coding paradigms in addition to utilizing objective cognitive developmental assessments of with normative data in South African children.

There are also several unmeasured contextual factors which could affect our findings. For example, although prenatal maternal and child nutrition are known to affect child neurobehavioral development, we lacked measures of prenatal maternal nutrition, prenatal maternal micronutrient deficiencies, prenatal and postnatal household food insecurity, and information regarding child nutrition. Additionally, prior studies have emphasized the importance of measuring childhood trauma, maternal stress, and social support within communities with several heterogeneous risk factors[3], these measures were not collected as part of the original NIH Safe Passage Study or our recent follow-up study. Therefore, we could not examine their effects or consider maternal social support as a potential moderator of resilient neurodevelopmental outcomes. There is also robust literature examining both postpartum depression (PPD) and the early mother-infant relation in shaping child outcomes. Two prior studies in South Africa demonstrated maternal intrusiveness and coerciveness mediated the association between maternal PPD and early childhood attachment[58 59]. In the current study, we did not collect data postnatally between birth and three years of age. Additionally, we did not collect postnatal information on maternal depression, anxiety, or stress. Therefore, due to this methodological limitation we cannot draw conclusions regarding the combined effect of the pre- and postnatal environment on child neurodevelopmental outcomes, nor can we assess potential interaction effects between pre- and postnatal maternal mood on child social-emotional and cognitive outcomes. Finally, our results may not generalize to all South African populations where HIV rates can be as high as 35% since our cohort only included one woman with HIV.

For peer review only - http://bmjopen.bmj.com/site/about/guidelines.xhtml

Strengths of the current analyses include our large sample size, evaluation of both cognitive and social-emotional outcomes within the same cohort, finally the detailed prospective collection of both depression and anxiety symptoms during pregnancy[31] in addition to other potential confounders.

Our results suggest comorbid prenatal maternal depression and chronic anxiety have a greater impact on child social-emotional and cognitive development than either condition alone or than comorbid prenatal maternal depression and transitory anxiety during pregnancy. These findings are supported by a robust literature within the DOHaD framework linking perturbations in the gestational environment to later neurodevelopmental or psychiatric sequelae. Our results also lend support for future intervention studies aimed at perinatal mental health interventions targeting maternal depressive and anxiety symptoms to improve long-term child social-emotional and cognitive developmental outcomes in low-resource communities.

For peer review only

For peer review only - http://bmjopen.bmj.com/site/about/guidelines.xhtml

Table 1 - Sociodemographic characteristics			
		Mean ± SD or N (%)	N and Percent Missing (%)
**Maternal Characteristics**			
Maternal Age (years)		25.26 ± 5.91	0 (0%)
Maternal Body Mass Index (BMI)			0 (0%)
	BMI <18.5 (kg/m2)	62 (10.33%)	
	BMI 18.5-25 (kg/m2)	282 (47%)	
	BMI 25-30 (kg/m2)	132 (22%)	
	BMI >30 (kg/m2)	124 (20.67%)	
Parity			0 (0%)
	Parity <1	218 (36.33%)	
	Parity =1	196 (32.67%)	
	Parity =2	103 (17.17%)	
	Parity >=3	83 (13.83%)	
Antenatal care visits			0 (0%)
	antenatal care visit <3	49 (8.17%)	
	antenatal care visit 3-6	389 (64.83%)	
	antenatal care visit >6	162 (27%)	
Cesarean Section		106 (17.70%)	1 (0.1%)
Education			0 (0%)
	Some primary school	43 (7.167%)	
	Some high school	405 (67.57%)	
	Completed high school	119 (19.83%)	
	Beyond high school	33 (5.5%)	
Married		290 (48.33%)	0 (0%)
Employed		172 (28.67%)	0 (0%)
Adjusted Household Crowding		1.56 ± 0.72	2 (0.33%)
Depression (Edinburgh >=13)		333 (55%)	0 (0%)
Anxiety (State-Trait Anxiety Inventory >=40)		115 (19.17%)	0 (0%)
Maternal Prenatal Alcohol Use Cluster Groups			0 (0%)
Non drinking group: 0 standard drinks/trimester		245 (46.93%)	
Moderate-high continuous drinking group:		122 (23.37%)	
	Standard drinks in Trimester 1	27 ± 39	
	Standard drinks in Trimester 2	17 ± 25	

For peer review only

Standard drinks in Trimester 3	9.4 ± 15	
Binge drinking events (≥4 drinks/day) Trimester 1	2.7 ± 4	
Binge drinking events (≥4 drinks/day) Trimester 2	1.7 ± 2.8	
Binge drinking events (≥4 drinks/day) Trimester 3	0.89 ± 1.7	
Low continuous drinking	26 (4.98%)	
Standard drinks in Trimester 1	1.4 ± 2.5	
Standard drinks in Trimester 2	4 ± 2.8	
Standard drinks in Trimester 3	0.62 ± 1.1	
Binge drinking events (≥4 drinks/day) Trimester 1	0.067 ± 0.25	
Binge drinking events (≥4 drinks/day) Trimester 2	0.30 ± 0.46	
Binge drinking events (≥4 drinks/day) Trimester 3	0 ± 0	
Quit early drinking	129 (24.71%)	
Standard drinks in Trimester 1	8.5 ± 6.5	
Standard drinks in Trimester 2	0.31 ± 0.87	
Standard drinks in Trimester 3	0.056 ± 0.31	
Binge drinking events (≥4 drinks/day) Trimester 1	0.84 ± 0.82	
Binge drinking events (≥4 drinks/day) Trimester 2	0 ± 0	
Binge drinking events (≥4 drinks/day) Trimester 3	0 ± 0	
Maternal Prenatal Tobacco Use		2 (0.33%)
Non smoking (0 cigarettes/trimester)	227 (37.97%)	
Moderate-high continuous smoking	132 (22.07%)	
Average Cigarettes in Trimester 1	45 ± 20	
Average Cigarettes in Trimester 2	50 ± 27	
Average Cigarettes in Trimester 3	48 ± 25	
Low continuous smoking	222 (37.12%)	
Average Cigarettes in Trimester 1	16 ± 9.4	
Average Cigarettes in Trimester 2	16 ± 9.7	
Average Cigarettes in Trimester 3	16 ± 10	
Quit early smoking	17 (2.84%)	
Average Cigarettes in Trimester 1	11 ± 7.5	
Average Cigarettes in Trimester 2	0.15 ± 0.32	
Average Cigarettes in Trimester 3	0.079 ± 0.11	
Raw Maternal Edinburgh Score	12.99 ± 5.73	0 (0%)
Raw Maternal State Anxiety Score	31.23 ± 10.24	0 (0%)
Raw Maternal Trait Anxiety Score	40.63 ± 10.63	0 (0%)
Depression - Trait Anxiety Groups		0 (0%)
No depression or trait anxiety	199 (33.17%)	
Depression alone	106 (17.67%)	
Trait anxiety alone	68 (11.33%)	
Depression and trait anxiety	227 (37.83%)	

For peer review only

Depression - State Anxiety Groups			0 (0%)
	No depression or state anxiety	248 (41.33%)	
	Depression alone	237 (39.50%)	
	State anxiety alone	19 (3.17%)	
	Depression and state anxiety	96 (16%)	
HIV status			
	Tested for HIV	600 (100%)	
	HIV positive	1 (0.1%)	
**Infant Characteristics**			
Infant Sex			0 (0%)
	Male	326 (54.33%)	
	Female	274 (45.67%)	
Gestational age at birth (weeks)		38.89 ± 2.03	0 (0%)
Infant Birth weight (grams)		2980.65 ± 564.65	2 (0.33%)
Follow-up age (months)		38.29 ± 2.96	0 (0%)
Adjusted follow-up age (months)		38.14 ± 0.016	0 (0%)

For peer review only

**Table 2. Neurodevelopmental Outcome Raw Scores and At-risk Groups**		
BSID-III Screening Test Language of Administration		
	English Only	177 (30%)
	Afrikaans Only	360 (62%)
	Mixed English and Afrikaans	48 (8%)
BSID-III Screening Test Scores		
	Gross motor	25.47 ± 4.54
	Fine motor	23.55 ± 4.08
	Cognitive	27.77 ± 4.99
	Problem solving	13.40 ± 6.77
	Receptive language	21.69 ± 5.10
	Expressive language	21.27 ± 3.78
At-Risk Categories		
	Gross motor	21 (4%)
	Fine motor	18 (3%)
	Cognitive	24 (4%)
	Receptive language	33 (6%)
	Expressive language	25 (4%)
Brief Infant Toddler Social-Emotional Assessment (BITSEA)		
	Social Emotional Problem	13.40 ± 6.77
	Competence	19.56 ± 2.07

For peer review only - http://bmjopen.bmj.com/site/about/guidelines.xhtml

At-Risk Categories		
	Social Emotional Problems	306 (51%)
	Social-Emotional Competence	30 (5%)

Figure Legends

Figure 1. Association between prenatal maternal depression, prenatal maternal anxiety, and child social-emotional problems measured by the BITSEA. Each line plot depicts the mean difference and their confidence interval for child social-emotional problems (x-axis) for each prenatal maternal mental health group (y-axis). Either no prenatal maternal depression and state anxiety or no prenatal maternal depression and trait anxiety were the reference groups. Models were adjusted for sex, gestational age at birth, age at follow-up, prenatal maternal alcohol use, prenatal maternal tobacco use, maternal employment status at delivery, maternal educational attainment at delivery, parity, and the household crowding index.

Figure 2. Association between prenatal maternal depression, prenatal maternal anxiety, and child cognitive development measured by the BSID-III Screening Test. Each line plot depicts the mean difference and their confidence interval for child cognitive development (x-axis) for each prenatal maternal mental health group (y-axis). Either no prenatal maternal depression and state anxiety or no prenatal maternal depression and trait anxiety were the reference groups. Models were adjusted for sex, gestational age at birth, age at follow-up, prenatal maternal alcohol use, prenatal maternal tobacco use, maternal employment status at delivery, maternal educational attainment at delivery, parity, and the household crowding index.

References

1. O'Donnell KJ, Meaney MJ. Fetal Origins of Mental Health: The Developmental Origins of Health and Disease Hypothesis. *Am J Psychiatry* 2017;174(4):319-28. doi: 10.1176/appi.ajp.2016.16020138 [published Online First: 2016/11/15]
2. Hofer MA. The Roots of Human Behavior: An Introduction to the Psychobiology of Early Development. San Francisco, CA: W. H. Freeman & Company 1981.
3. Stein DJ, Koen N, Donald KA, et al. Investigating the psychosocial determinants of child health in Africa: The Drakenstein Child Health Study. *J Neurosci Methods* 2015;252:27-35. doi: 10.1016/j.jneumeth.2015.03.016 [published Online First: 2015/03/24]
4. Herba CM, Glover V, Ramchandani PG, et al. Maternal depression and mental health in early childhood: an examination of underlying mechanisms in low-income and middle-income countries. *Lancet Psychiatry* 2016;3(10):983-92. doi: 10.1016/S2215-0366(16)30148-1 [published Online First: 2016/09/22]

For peer review only

5. Monk C, Lugo-Candelas C, Trumpff C. Prenatal Developmental Origins of Future Psychopathology: Mechanisms and Pathways. *Annu Rev Clin Psychol* 2019;15:317-44. doi: 10.1146/annurev-clinpsy-050718-095539 [published Online First: 2019/02/24]
6. Madigan S, Oatley H, Racine N, et al. A Meta-Analysis of Maternal Prenatal Depression and Anxiety on Child Socioemotional Development. *J Am Acad Child Adolesc Psychiatry* 2018;57(9):645-57 e8. doi: 10.1016/j.jaac.2018.06.012
7. King S, Laplante DP. The effects of prenatal maternal stress on children's cognitive development: Project Ice Storm. *Stress* 2005;8(1):35-45. doi: 10.1080/10253890500108391
8. Huizink AC, Robles de Medina PG, Mulder EJ, et al. Stress during pregnancy is associated with developmental outcome in infancy. *J Child Psychol Psychiatry* 2003;44(6):810-8. doi: 10.1111/1469-7610.00166 [published Online First: 2003/09/10]
9. Leis JA, Heron J, Stuart EA, et al. Associations between maternal mental health and child emotional and behavioral problems: does prenatal mental health matter? *J Abnorm Child Psychol* 2014;42(1):161-71. doi: 10.1007/s10802-013-9766-4
10. O'Connor TG, Heron J, Golding J, et al. Maternal antenatal anxiety and children's behavioural/emotional problems at 4 years. Report from the Avon Longitudinal Study of Parents and Children. *Br J Psychiatry* 2002;180:502-8.
11. O'Donnell KJ, Glover V, Barker ED, et al. The persisting effect of maternal mood in pregnancy on childhood psychopathology. *Dev Psychopathol* 2014;26(2):393-403. doi: 10.1017/S0954579414000029
12. Loomans EM, van der Stelt O, van Eijsden M, et al. Antenatal maternal anxiety is associated with problem behaviour at age five. *Early Hum Dev* 2011;87(8):565-70. doi: 10.1016/j.earlhumdev.2011.04.014
13. Rice F, Harold GT, Boivin J, et al. The links between prenatal stress and offspring development and psychopathology: disentangling environmental and inherited influences. *Psychol Med* 2010;40(2):335-45. doi: 10.1017/S0033291709005911
14. Ronald A, Pennell CE, Whitehouse AJ. Prenatal Maternal Stress Associated with ADHD and Autistic Traits in early Childhood. *Front Psychol* 2010;1:223. doi: 10.3389/fpsyg.2010.00223 [published Online First: 2010/01/01]
15. Lereya ST, Wolke D. Prenatal family adversity and maternal mental health and vulnerability to peer victimisation at school. *J Child Psychol Psychiatry* 2013;54(6):644-52. doi: 10.1111/jcpp.12012
16. Rogers A, Obst S, Teague SJ, et al. Association Between Maternal Perinatal Depression and Anxiety and Child and Adolescent Development: A Meta-analysis. *JAMA Pediatr* 2020;174(11):1082-92. doi: 10.1001/jamapediatrics.2020.2910 [published Online First: 2020/09/15]
17. Van den Bergh BRH, van den Heuvel MI, Lahti M, et al. Prenatal developmental origins of behavior and mental health: The influence of maternal stress in pregnancy. *Neurosci Biobehav Rev* 2017 doi: 10.1016/j.neubiorev.2017.07.003 [published Online First: 2017/08/02]
18. Evans LM, Myers MM, Monk C. Pregnant women's cortisol is elevated with anxiety and depression - but only when comorbid. *Arch Womens Ment Health* 2008;11(3):239-48. doi: 10.1007/s00737-008-0019-4 [published Online First: 2008/05/22]

For peer review only - http://bmjopen.bmj.com/site/about/guidelines.xhtml

19. Leff Gelman P, Mancilla-Herrera I, Flores-Ramos M, et al. The cytokine profile of women with severe anxiety and depression during pregnancy. *BMC Psychiatry* 2019;19(1):104. doi: 10.1186/s12888-019-2087-6 [published Online First: 2019/04/05]
20. Osborne LM, Yenokyan G, Fei K, et al. Innate immune activation and depressive and anxious symptoms across the peripartum: An exploratory study. *Psychoneuroendocrinology* 2019;99:80-86. doi: 10.1016/j.psyneuen.2018.08.038 [published Online First: 2018/09/09]
21. Osborne LM, Brar A, Klein SL. The role of Th17 cells in the pathophysiology of pregnancy and perinatal mood and anxiety disorders. *Brain Behav Immun* 2019;76:7-16. doi: 10.1016/j.bbi.2018.11.015 [published Online First: 2018/11/23]
22. Eshetu EB, Woldesenbet SA. Are there particular social determinants of health for the world's poorest countries? *African Health Sciences* 2011;11(1):108 - 15.
23. Davies T, Rahman A, Lund C. Psychotherapy for perinatal mental disorders in low- and middle-income countries. In Global Mental Health in Practice, Global Mental Health and Psychotherapy: Academic Press, 2019:301 - 19.
24. Gordon S, Rotheram-Fuller E, Rezvan P, et al. Maternal depressed mood and child development over the first five years of life in South Africa. *J Affect Disord* 2021;294:346-56. doi: 10.1016/j.jad.2021.07.027 [published Online First: 2021/07/28]
25. Heyningen TV, Myer L, Onah M, et al. Antenatal depression and adversity in urban South Africa. *J Affect Disord* 2016;203:121-29. doi: 10.1016/j.jad.2016.05.052 [published Online First: 2016/06/11]
26. Redinger S, Norris SA, Pearson RM, et al. First trimester antenatal depression and anxiety: prevalence and associated factors in an urban population in Soweto, South Africa. *J Dev Orig Health Dis* 2018;9(1):30-40. doi: 10.1017/S204017441700071X [published Online First: 2017/09/08]
27. Redinger S, Pearson RM, Houle B, et al. Antenatal depression and anxiety across pregnancy in urban South Africa. *J Affect Disord* 2020;277:296-305. doi: 10.1016/j.jad.2020.08.010 [published Online First: 2020/08/29]
28. Drysdale RE, Slemming W, Makusha T, et al. Father involvement, maternal depression and child nutritional outcomes in Soweto, South Africa. *Matern Child Nutr* 2021;17 Suppl 1:e13177. doi: 10.1111/mcn.13177 [published Online First: 2021/07/10]
29. Ramchandani PG, Richter LM, Norris SA, et al. Maternal prenatal stress and later child behavioral problems in an urban South African setting. *J Am Acad Child Adolesc Psychiatry* 2010;49(3):239-47. [published Online First: 2010/04/23]
30. Donald KA, Wedderburn CJ, Barnett W, et al. Risk and protective factors for child development: An observational South African birth cohort. *PLoS Med* 2019;16(9):e1002920. doi: 10.1371/journal.pmed.1002920 [published Online First: 2019/09/29]
31. Dukes KA, Burd L, Elliott AJ, et al. The Safe Passage Study: Design, Methods, Recruitment, and Follow-Up Approach. *Paediatr Perinat Ep* 2014;28(5):455-65. doi: 10.1111/ppe.12136
32. Dukes K, Tripp T, Petersen J, et al. A modified Timeline Followback assessment to capture alcohol exposure in pregnant women: Application in the Safe Passage Study. *Alcohol* 2017;62:17-27. doi: 10.1016/j.alcohol.2017.02.174

For peer review only - http://bmjopen.bmj.com/site/about/guidelines.xhtml

33. Pini N, Shuffrey, LC., Lucchini, M., Sania, A., Nelson, ME., Odendaal, HJ., Fifer, WP., Myers, MM., Elliott, AJ. Characterization of Alcohol Consumption during Pregnancy in the Safe Passage Study. Conf Proc IEEE Eng Med Biol Soc. Berlin, Germany.
34. Shuffrey LC, Myers MM, Isler JR, et al. Association Between Prenatal Exposure to Alcohol and Tobacco and Neonatal Brain Activity: Results From the Safe Passage Study. *JAMA Netw Open* 2020;3(5):e204714. doi: 10.1001/jamanetworkopen.2020.4714 [published Online First: 2020/05/13]
35. Lawrie TA, Hofmeyr GJ, de Jager M, et al. Validation of the Edinburgh Postnatal Depression Scale on a cohort of South African women. *S Afr Med J* 1998;88(10):1340-4. [published Online First: 1998/11/10]
36. Alvarado R, Jadresic E, Guajardo V, et al. First validation of a Spanish-translated version of the Edinburgh postnatal depression scale (EPDS) for use in pregnant women. A Chilean study. *Arch Womens Ment Health* 2015;18(4):607-12. doi: 10.1007/s00737-014-0466-z [published Online First: 2014/10/11]
37. Hartley M, Tomlinson M, Greco E, et al. Depressed mood in pregnancy: prevalence and correlates in two Cape Town peri-urban settlements. *Reprod Health* 2011;8:9. doi: 10.1186/1742-4755-8-9 [published Online First: 2011/05/04]
38. Spielberger CD. Manual for the State-Trait Anxiety Inventory: STAI. Palo Alto, CA: Consulting Psychologists Press, 1983.
39. Pretorius TB, Norman AM. Psychometric Data on the Statistics Anxiety Scale for a Sample of South-African Students. *Educational and Psychological Measurement* 1992;52(4):933-37. doi: Doi 10.1177/0013164492052004015
40. Rademeyer V, Jacklin L. A study to evaluate the performance of black South African urban infants on the Bayley Scales of Infant Development III. *South African Journal of Child Health* 2013;7(2):54-59.
41. Ballot DE, Ramdin T, Rakotsoane D, et al. Use of the Bayley Scales of Infant and Toddler Development, Third Edition, to Assess Developmental Outcome in Infants and Young Children in an Urban Setting in South Africa. *Int Sch Res Notices* 2017;2017:1631760. doi: 10.1155/2017/1631760 [published Online First: 2017/08/25]
42. Gowan B, Carter MJ, Carter AS. ITSEA/BITSEA: Infant-Toddler and Brief Infant-Toddler Social and Emotional Assessment. *The Psychological Corporation* 2006
43. Kruizinga I, Visser JC, van Batenburg-Eddes T, et al. Screening for autism spectrum disorders with the brief infant-toddler social and emotional assessment. *PLoS One* 2014;9(5):e97630. doi: 10.1371/journal.pone.0097630 [published Online First: 2014/05/24]
44. Sang S, Nelson, JA. The effect of siblings on children's social skills and perspective taking. *Inf Child Dev* 2017:26:e2023.
45. Downey DB CD, Yucel D. Number of Siblings and Social Skills Revisited Among American Fifth Graders. *Journal of Family Issues* 2015:36(2):273-96. doi: 10.1177/0192513X13507569
46. Ghassabian A, Albert PS, Hornig M, et al. Gestational cytokine concentrations and neurocognitive development at 7 years. *Transl Psychiat* 2018;8 doi: ARTN 64
10.1038/s41398-018-0112-z
47. Ruiz RJ, Avant KC. Effects of maternal prenatal stress on infant outcomes - A synthesis of the literature. *Adv Nurs Sci* 2005;28(4):345-55. doi: Doi 10.1097/00012272-200510000-00006

For peer review only - http://bmjopen.bmj.com/site/about/guidelines.xhtml

48. Short SJ, Lubach GR, Karasin AI, et al. Maternal influenza infection during pregnancy impacts postnatal brain development in the rhesus monkey. *Biol Psychiatry* 2010;67(10):965-73. doi: 10.1016/j.biopsych.2009.11.026 [published Online First: 2010/01/19]
49. Paolicelli RC, Bolasco G, Pagani F, et al. Synaptic pruning by microglia is necessary for normal brain development. *Science* 2011;333(6048):1456-8. doi: 10.1126/science.1202529 [published Online First: 2011/07/23]
50. Bergdolt L, Dunaevsky A. Brain changes in a maternal immune activation model of neurodevelopmental brain disorders. *Prog Neurobiol* 2019;175:1-19. doi: 10.1016/j.pneurobio.2018.12.002 [published Online First: 2018/12/28]
51. Meyer U. Neurodevelopmental Resilience and Susceptibility to Maternal Immune Activation. *Trends Neurosci* 2019;42(11):793-806. doi: 10.1016/j.tins.2019.08.001 [published Online First: 2019/09/09]
52. Patterson PH. Maternal infection: window on neuroimmune interactions in fetal brain development and mental illness. *Curr Opin Neurobiol* 2002;12(1):115-8. doi: 10.1016/s0959-4388(02)00299-4 [published Online First: 2002/02/28]
53. Bland ST, Beckley JT, Young S, et al. Enduring consequences of early-life infection on glial and neural cell genesis within cognitive regions of the brain. *Brain Behav Immun* 2010;24(3):329-38. doi: 10.1016/j.bbi.2009.09.012 [published Online First: 2009/09/29]
54. Money KM, Barke TL, Serezani A, et al. Gestational diabetes exacerbates maternal immune activation effects in the developing brain. *Mol Psychiatry* 2018;23(9):1920-28. doi: 10.1038/mp.2017.191 [published Online First: 2017/09/28]
55. Bilbo SD, Schwarz JM. Early-life programming of later-life brain and behavior: a critical role for the immune system. *Front Behav Neurosci* 2009;3:14. doi: 10.3389/neuro.08.014.2009 [published Online First: 2009/09/10]
56. Buss C, Davis EP, Shahbaba B, et al. Maternal cortisol over the course of pregnancy and subsequent child amygdala and hippocampus volumes and affective problems. *P Natl Acad Sci USA* 2012;109(20):E1312-E19. doi: 10.1073/pnas.1201295109
57. Yehuda R, Lehrner A. Intergenerational transmission of trauma effects: putative role of epigenetic mechanisms. *World Psychiatry* 2018;17(3):243-57. doi: 10.1002/wps.20568 [published Online First: 2018/09/08]
58. Cooper PJ, Tomlinson M, Swartz L, et al. Post-partum depression and the mother-infant relationship in a South African peri-urban settlement. *Br J Psychiatry* 1999;175:554-8. doi: 10.1192/bjp.175.6.554 [published Online First: 2000/05/02]
59. Tomlinson M, Cooper P, Murray L. The mother-infant relationship and infant attachment in a South African peri-urban settlement. *Child Dev* 2005;76(5):1044-54. doi: 10.1111/j.1467-8624.2005.00896.x [published Online First: 2005/09/10]

For peer review only

For peer review only - http://bmjopen.bmj.com/site/about/guidelines.xhtml

Contributors: WPF and HJO acquired funding for and designed the work. LCS, AS, NHB, ML, YR, PES, HJO, and WPF conceptualized the work. MP, PES, CDP, and HJO acquired the data. LCS, NHB, and WPF led in data collection oversight and quality control. AS, LCS, and YR analyzed the data. All authors contributed to the interpretation of data and drafting the work and revising it critically for important intellectual content. All authors approved the final manuscript and agree to be accountable for all aspects of the work.

Acknowledgements: We would like to acknowledge Lucy Brink, J. David Nugent, and Daianna Rodriguez for their database and administrative support. We would also like to thank all of the families who participated in this research study for making this work possible.

Ethics statements

Patient consent for publication

Not applicable.

Ethics approval

Ethics approval was received from the Institutional Review Boards and ethics review committees at Stellenbosch University (N16-08-101 and N06-10-210) and the New York State Psychiatric Institute (5338). All participants provided informed written consent at both time points (prenatal and postnatal) before inclusion in the study.

For peer review only

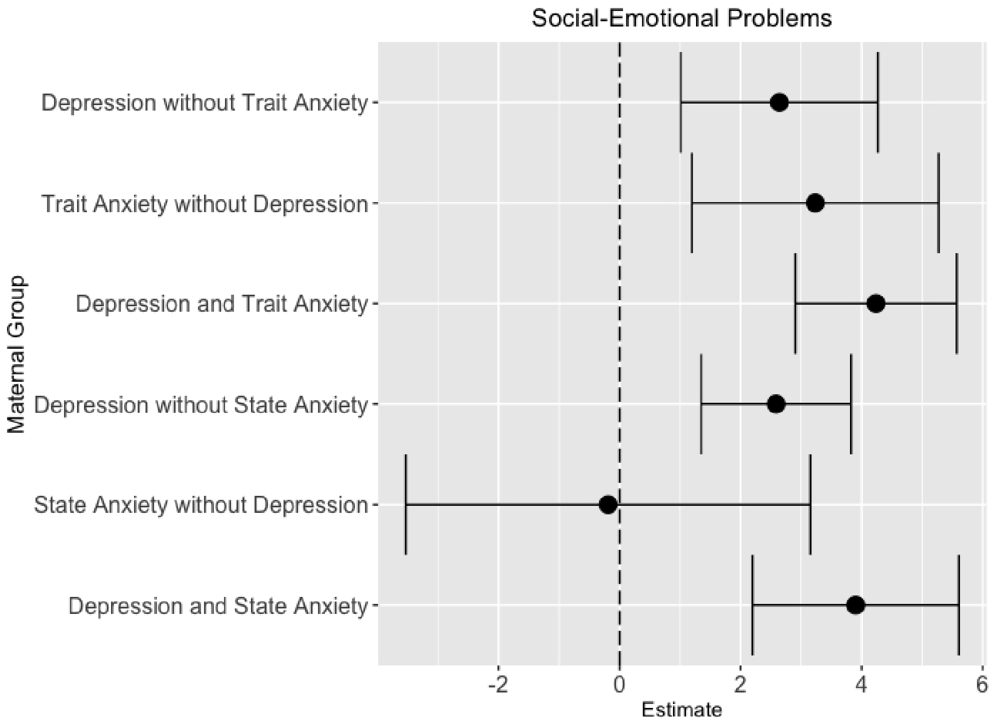

Figure 1. Association between prenatal maternal depression, prenatal maternal anxiety, and child social-emotional problems measured by the BITSEA. Each line plot depicts the mean difference and their confidence interval for child social-emotional problems (x-axis) for each prenatal maternal mental health group (y-axis). Either no prenatal maternal depression and state anxiety or no prenatal maternal depression and trait anxiety were the reference groups. Models were adjusted for sex, gestational age at birth, age at follow-up, prenatal maternal alcohol use, prenatal maternal tobacco use, maternal employment status at delivery, maternal educational attainment at delivery, parity, and the household crowding index.

247x181mm (72 x 72 DPI)

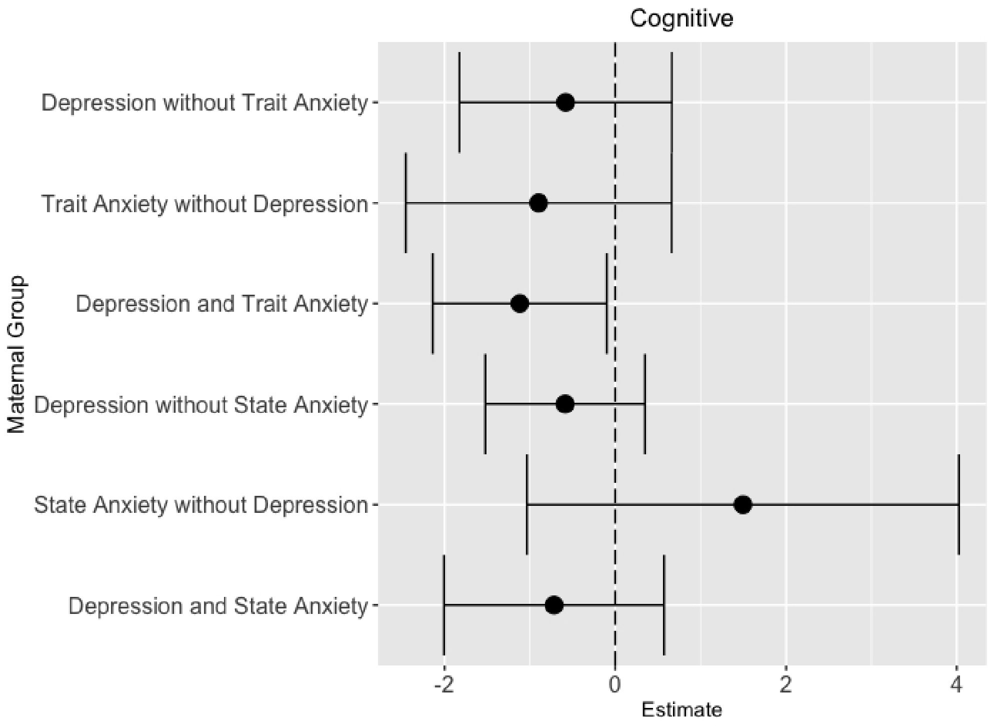

Figure 2. . Association between prenatal maternal depression, prenatal maternal anxiety, and child cognitive development measured by the BSID-III Screening Test. Each line plot depicts the mean difference and their confidence interval for child cognitive development (x-axis) for each prenatal maternal mental health group (y-axis). Either no prenatal maternal depression and state anxiety or no prenatal maternal depression and trait anxiety were the reference groups. Models were adjusted for sex, gestational age at birth, age at follow-up, prenatal maternal alcohol use, prenatal maternal tobacco use, maternal employment status at delivery, maternal educational attainment at delivery, parity, and the household crowding index.

247x181mm (72 x 72 DPI)

STROBE Statement—Checklist of items that should be included in reports of ***cohort studies***

	Item No	Recommendation	Page No
**Title and abstract**	1	(*a*) Indicate the study's design with a commonly used term in the title or the abstract	2
		(*b*) Provide in the abstract an informative and balanced summary of what was done and what was found	3, 4
**Introduction**			
Background/rationale	2	Explain the scientific background and rationale for the investigation being reported	5, 6
Objectives	3	State specific objectives, including any prespecified hypotheses	6
**Methods**			
Study design	4	Present key elements of study design early in the paper	7 - 9
Setting	5	Describe the setting, locations, and relevant dates, including periods of recruitment, exposure, follow-up, and data collection	7 - 9
Participants	6	(*a*) Give the eligibility criteria, and the sources and methods of selection of participants. Describe methods of follow-up	7 - 9
		(*b*) For matched studies, give matching criteria and number of exposed and unexposed	N/A
Variables	7	Clearly define all outcomes, exposures, predictors, potential confounders, and effect modifiers. Give diagnostic criteria, if applicable	9 - 10
Data sources/ measurement	8*	For each variable of interest, give sources of data and details of methods of assessment (measurement). Describe comparability of assessment methods if there is more than one group	7 - 10
Bias	9	Describe any efforts to address potential sources of bias	10
Study size	10	Explain how the study size was arrived at	11
Quantitative variables	11	Explain how quantitative variables were handled in the analyses. If applicable, describe which groupings were chosen and why	9 - 10
Statistical methods	12	(*a*) Describe all statistical methods, including those used to control for confounding	9 - 10
		(*b*) Describe any methods used to examine subgroups and interactions	9 - 10
		(*c*) Explain how missing data were addressed	10
		(*d*) If applicable, explain how loss to follow-up was addressed	10
		(*e*) Describe any sensitivity analyses	N/A
**Results**			
Participants	13*	(a) Report numbers of individuals at each stage of study—eg numbers potentially eligible, examined for eligibility, confirmed eligible, included in the study, completing follow-up, and analysed	11
		(b) Give reasons for non-participation at each stage	11
		(c) Consider use of a flow diagram	
Descriptive data	14*	(a) Give characteristics of study participants (eg demographic, clinical, social) and information on exposures and potential confounders	9, 11 - 13
		(b) Indicate number of participants with missing data for each variable of interest	10
		(c) Summarise follow-up time (eg, average and total amount)	
Outcome data	15*	Report numbers of outcome events or summary measures over time	11

For peer review only - http://bmjopen.bmj.com/site/about/guidelines.xhtml

Main results	16	(*a*) Give unadjusted estimates and, if applicable, confounder-adjusted estimates and their precision (eg, 95% confidence interval). Make clear which confounders were adjusted for and why they were included<br>(*b*) Report category boundaries when continuous variables were categorized<br>(*c*) If relevant, consider translating estimates of relative risk into absolute risk for a meaningful time period	11 - 13<br><br>N/A
Other analyses	17	Report other analyses done—eg analyses of subgroups and interactions, and sensitivity analyses	11 - 13
**Discussion**			
Key results	18	Summarise key results with reference to study objectives	14
Limitations	19	Discuss limitations of the study, taking into account sources of potential bias or imprecision. Discuss both direction and magnitude of any potential bias	17
Interpretation	20	Give a cautious overall interpretation of results considering objectives, limitations, multiplicity of analyses, results from similar studies, and other relevant evidence	17
Generalisability	21	Discuss the generalisability (external validity) of the study results	17
**Other information**			
Funding	22	Give the source of funding and the role of the funders for the present study and, if applicable, for the original study on which the present article is based	1

*Give information separately for exposed and unexposed groups.

Note: An Explanation and Elaboration article discusses each checklist item and gives methodological background and published examples of transparent reporting. The STROBE checklist is best used in conjunction with this article (freely available on the Web sites of PLoS Medicine at http://www.plosmedicine.org/, Annals of Internal Medicine at http://www.annals.org/, and Epidemiology at http://www.epidem.com/). Information on the STROBE Initiative is available at http://www.strobe-statement.org.

For peer review only
